# Strong Photon‐Magnon Coupling Using a Lithographically Defined Organic Ferrimagnet

**DOI:** 10.1002/advs.202310032

**Published:** 2024-01-26

**Authors:** Qin Xu, Hil Fung Harry Cheung, Donley S. Cormode, Tharnier O. Puel, Srishti Pal, Huma Yusuf, Michael Chilcote, Michael E. Flatté, Ezekiel Johnston‐Halperin, Gregory D. Fuchs

**Affiliations:** ^1^ Department of Physics Cornell University Ithaca NY 14853 USA; ^2^ Department of Physics The Ohio State University Columbus OH 43210 USA; ^3^ Department of Physics and Astronomy University of Iowa Iowa City IA 52242 USA; ^4^ School of Applied and Engineering Physics Cornell University Ithaca NY 14853 USA

**Keywords:** cavity magnonics, hybrid quantum system, lithographically defined low damping organic ferrimagnet, non‐uniform magnon modes, strong coupling, vanadium tetracyanoethylene

## Abstract

A cavity‐magnonic system composed of a superconducting microwave resonator coupled to a magnon mode hosted by the organic‐based ferrimagnet vanadium tetracyanoethylene (V[TCNE]_*x*_) is demonstrated. This work is motivated by the challenge of scalably integrating a low‐damping magnetic system with planar superconducting circuits. V[TCNE]_*x*_ has ultra‐low intrinsic damping, can be grown at low processing temperatures on arbitrary substrates, and can be patterned via electron beam lithography. The devices operate in the strong coupling regime, with a cooperativity exceeding 1000 for coupling between the Kittel mode and the resonator mode at T≈0.4 K, suitable for scalable quantum circuit integration. Higher‐order magnon modes are also observed with much narrower linewidths than the Kittel mode. This work paves the way for high‐cooperativity hybrid quantum devices in which magnonic circuits can be designed and fabricated as easily as electrical wires.

## Graphical abstract

Strong coupling between superconducting microwave resonator photons and the magnons of an organic‐based magnetic material is reported. The results also show that this material has ultra‐low magnetic dissipation that is competitive with the best solid‐state materials at low temperature. The precise integration of these two systems is promising for flexible and scalable magnonic circuit elements that operate in the quantum limit.

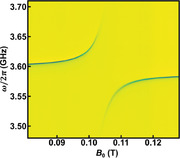

## 1 Introduction

Hybrid quantum systems are attractive for emerging quantum technologies because they take advantage of the distinct properties of the constituent excitations.^[^ ^1^, ^2^ ^]^ This is important because no single quantum system is ideal for every task, e.g., scalable quantum information processing, quantum sensing, long‐lived quantum memory, and long‐range quantum communication all have different requirements. Some hybrid systems that have been explored are microwave photons hybridized with spins,^[^ ^3^, ^4^, ^5^, ^6^, ^7^, ^8^, ^9^ ^]^ optical photons hybridized with atomic degrees of freedom,^[^ ^10^, ^11^, ^12^ ^]^ and superconducting qubits hybridized with phonons.^[^ ^13^ ^]^ In creating hybrid systems, it is advantageous to operate in the strong‐coupling, low‐loss regime, where the relaxation rates of the two distinct quantum systems are exceeded by the coupling rate between them. This allows the hybrid system to operate as a quantum interconnect, wherein quantum information can be passed from one excitation to another.^[^ ^2^ ^]^ Thus, a central challenge is to couple distinct quantum systems strongly, with all elements maintaining long coherence times. An equally critical challenge is to fabricate the hybrid quantum devices using scalable and integrable approaches so that their engineered properties can be used in applications.

Combining magnons (quantized spin waves) and microwave photons into a hybrid system has been analyzed theoretically^[^ ^14^, ^15^, ^16^ ^]^ and studied experimentally.^[^ ^17^, ^18^ ^]^ One attraction of this combination is that it can harness the unique properties of magnetic materials.^[^ ^14^, ^19^, ^20^, ^21^ ^]^ One example is that magnetic materials break time‐reversal symmetry, providing an opportunity to engineer nonreciprocal devices that are interesting for quantum applications.^[^ ^22^, ^23^ ^]^ Magnon‐cavity interactions have shown nonreciprocal behavior in suitably designed structures.^[^ ^24^, ^25^ ^]^ Additionally, magnetic materials are promising for coupling to long‐lived quantum spin systems,^[^ ^26^, ^27^, ^28^ ^]^ potentially enabling quantum interconnects between important quantum technologies based on microwave photons and on color center spins.^[^ ^29^ ^]^

The challenge of integrating a microwave resonator with a low‐damping magnetic material that can be lithographically patterned has limited the scalability of hybrid photon‐magnon systems. Landmark initial demonstrations used large (millimeter‐scale) crystals of the ferrimagnetic insulator yttrium iron garnet (YIG) because of its record‐low damping, even at temperatures below 1 K for bulk crystals.^[^ ^17^, ^18^ ^]^ These efforts used either 3D copper microwave cavities^[^ ^18^, ^30^, ^31^, ^32^ ^]^ or a planar waveguide cavity.^[^ ^17^, ^33^ ^]^ Micron scale YIG films have also been integrated with three dimensional^[^ ^34^, ^35^ ^]^ and planar^[^ ^36^, ^37^ ^]^ cavities. Another approach was the integration of ferromagnetic alloys that can be directly grown and patterned on a planar cavity without the need for high‐temperature processing or lattice matching, which substantially enhances the scalability of this hybrid system.^[^ ^38^, ^39^, ^40^ ^]^ Unfortunately, these ferromagnetic materials have larger intrinsic magnetic damping than high‐quality bulk YIG crystals.

In this work, we demonstrate an alternate pathway to a strongly‐coupled hybrid magnonic system in which a low‐damping magnetic film is patterned directly on a superconducting resonator under gentle growth conditions. We develop a process to integrate lithographically‐patterned films of the organic‐based ferrimagnet vanadium tetracyanoethylene (V[TCNE]_*x*_) with a planar superconducting microwave resonator. V[TCNE]_*x*_ has a typical Gilbert damping coefficient in the range α = (4 − 20) × 10^−5^,^[^ ^41^, ^42^, ^43^ ^]^ which is comparable to YIG bulk crystals and high‐quality YIG films grown on lattice matched substrates.^[^ ^44^, ^45^ ^]^ We demonstrate strong coupling between V[TCNE]_*x*_ magnons and resonator photons in two devices (3.6 and 9.2 GHz) with a cooperativity as high as ≈1000. This is critically enabling for integration and scaling, permitting future designs in which magnonic waveguides can be tailored as couplers or can mediate interactions between different quantum excitations in a planar superconducting circuit. Focusing on the 3.6 GHz device, we present a detailed microwave transmission spectrum that reveals not only the expected avoided level crossing of the resonator mode and the uniform magnon mode (the Kittel mode, or simply magnon mode unless otherwise stated) but also the resonator mode is hybridized with a discrete spectrum of higher‐order magnon modes. These higher‐order magnon modes show a much narrower linewidth than the uniform mode and, as expected, they have a lower coupling to the resonator comparing to the uniform mode. Finally, we study the relaxation rate of the hybrid system as a function of the system detuning in the frequency domain and the time domain. This work paves the way for future hybrid magnonic quantum systems by establishing an integrated and scalable platform enabling arbitrary design of the magnonic elements.

## 2 Results and Discussion

The Hamiltonian describing a magnon mode coupled to a resonator mode is ref. [14, 18, 30, 39]:

(1) $$H_{0}/\hbar =\omega_{r}{\hat{a}}^{†}\hat{a}+\omega_{m}\left(B_{0}\right){\hat{b}}^{†}\hat{b}+g\left({\hat{b}}^{†}\hat{a}+\hat{b}{\hat{a}}^{†}\right)$$

where the first two terms describe, respectively, the energy of resonator photons and magnons at a static field *B*_0_, while the third term describes the photon‐magnon coupling. We have used ${\hat{a}}^{†}$ and $\hat{a}$ to describe the creation and annihilation of resonator photons, and ${\hat{b}}^{†}$ and $\hat{b}$ to describe the creation and annihilation of magnons. The collective coupling rate between the resonator photons and magnons is estimated relative to the single spin coupling rate *g*_*s*_ as [18] $g=g_{s}\sqrt{N}$, where *N* is the number of spins within the magnetic material coupled to the resonator.

To design a hybrid system that is useful for quantum circuit integration, we desire the hybrid system to operate in the strong‐coupling, low‐loss regime in which both the resonator damping rate κ_*r*_ and the magnon damping rate κ_*m*_ are smaller than *g*. It is also useful to parameterize the system in terms of its cooperativity, $C=4g^{2}/\kappa_{r}\kappa_{m}$, which is a dimensionless measure of system coherence that exceeds one when the two systems are strongly coupled.^[^ ^18^ ^]^ For such a system, when we tune the system into resonance, the excitations are best described as hybrids of resonator photons and magnons. While both κ_*r*_ and κ_*m*_ are separately important, C is an important figure‐of‐merit for hybrid systems. For example, in optomechanical cooling, the cooling power is proportional to C.^[^ ^46^ ^]^

First, we discuss the requirements of the magnetic material. Low material‐dependent magnetic (Gilbert) damping is critical for minimizing κ_*m*_. Additionally, it is a major materials challenge to precisely pattern and integrate low‐damping magnetic material with the superconducting resonator. For this purpose V[TCNE]_*x*_ is advantageous because it can be grown on nearly any substrate without the need for high temperature processing,^[^ ^47^, ^48^, ^49^, ^50^ ^]^ giving it wide substrate compatibility. Moreover, it can be patterned using electron beam lithography and lift‐off techniques without compromising the ultra‐low damping or introducing larger linewidth,^[^ ^41^, ^51^ ^]^ which overcomes the challenges of working with bulk‐grown crystals and thus has considerable advantages for scaling and integration. Another unique property of V[TCNE]_*x*_ is its low saturation magnetization of μ_0_ *M*_*s*_ ≈ 10 mT.^[^ ^41^, ^52^, ^53^ ^]^ Other physical properties of V[TCNE]_*x*_ have been extensively investigated^[^ ^42^, ^52^, ^53^, ^54^, ^55^, ^56^, ^57^, ^58^, ^59^ ^]^. Because V[TCNE]_*x*_  is air sensitive, we encapsulate it with epoxy and a coverslip to prevent air exposure while we handle the device. Previous studies have shown that this encapsulation will extend the lifetime of the V[TCNE]_*x*_ under ambient conditions from less than 1 hour to over 30 days^[^ ^42^, ^54^ ^]^ and it is expected to last indefinitely in either cryogenic conditions or refrigerated inert‐gas storage.^[^ ^42^ ^]^

**Figure 1:**
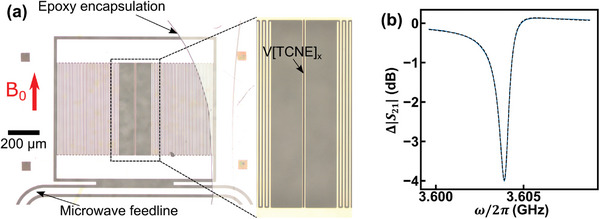
a) Microscope image of the 3.6 GHz resonator after V[TCNE]_*x*_ was deposited on its inductor wire. The V[TCNE]_*x*_ can be seen as a dark gray strip on the bright Nb wire at the center of the structure. The curved line is the border of the encapsulating epoxy. b) Transmission spectrum (blue) and fit (black dashed curve) at 0.0809 T when the device is at 0.43 K.

For the microwave resonator, we select a lumped‐element design consisting of a planar interdigitated capacitor that is shorted by a narrow inductor wire (**Figure** **1**a). This device is structurally similar to a transmon qubit that has a capacitor and a narrow inductor, except our structure does not include a Josephson junction as a part of the inductor. Instead, our narrow inductor wire efficiently couples resonator excitations to the magnon mode when the magnetic material is patterned directly on the wire surface. The resonator mode concentrates the oscillating current through the wire, generating an Oersted magnetic field that excites the spins.^[^ ^8^, ^39^, ^60^ ^]^ The resonator is capacitively coupled to a coplanar microwave feedline that we use to excite and detect the coupled resonator‐magnon system. Microwave electromagnetic simulations, the device fabrication procedure, V[TCNE]_*x*_ deposition details, and additional details of estimating the coupling strength are available in the Experimental Section and Section SI and SII (Supporting Information).

We start the electrical characterization of the device at 0.0809 T, a magnetic field at which the resonator mode and magnon mode is strongly detuned (we will see that it is by 7.2 × *g*). We measure the microwave transmission through the feedline using a vector network analyzer (VNA) at sample temperature *T* = 0.43 K. The result is shown in Figure 1b. The transmission coefficient of a microwave resonator coupled to a transmission line can be modeled as ref. [61]

(2) $$|S_{21}\left(\omega \right)|=\left|a\times \left(1-\frac{(Q_{l}/|Q_{c}\left|\right)e^{i\phi }}{1+2iQ_{l}\left(\omega /\omega_{res}-1\right)}\right)\right|$$

where *a* is the attenuation coefficient, ω_*res*_ ≈ ω_*r*_ is the mode resonance frequency and *Q*_*l*_ is the loaded quality‐factor that is used to calculate the total damping rate via κ_*l*_ = ω_*res*_/*Q*_*l*_. |*Q*_*c*_| is the magnitude of the coupling quality‐factor that parameterizes the loss of photons from the resonator to the waveguide and ϕ is the phase of *Q*_*c*_.

Fitting to Equation (2) reveals *Q*_*l*_ = 4302, |*Q*_*c*_| = 11200, and ω_*r*_/2π = 3.604 GHz. Since these measurements are far detuned from the magnon resonance, the measured damping rate is approximately the resonator damping κ_*r*_ = ω_*r*_/*Q*_*l*_ = 2π × 0.8377 MHz. As discussed below, we find that ω_*r*_ and κ_*r*_ have a weak but non‐zero magnetic field dependence. Using this measurement and another measurement at a magnetic field far above the magnetic resonance, we estimate κ_*r*_/2π to be 0.902(32) MHz at the resonance field *B*_*res*_ where ω_*m*_(*B*_*res*_) = ω_*r*_ (see the Supporting Information for details).

Next we tune the external magnetic field closer to the avoided level crossing between the microwave resonator mode and the uniform magnon (Kittel) mode to study the coupling between the two systems. The coupled eigenfrequencies are ref. [17]

(3) $$\omega_{\pm }=\omega_{r}+\Delta /2\pm \sqrt{\Delta^{2}+4g^{2}}/2$$

where Δ = ω_*m*_(*B*_0_) − ω_*r*_ is the system detuning, and the magnon frequency is $\omega_{m}\left(B_{0}\right)=\gamma \sqrt{B_{0}\left(B_{0}+\mu_{0}M_{\text{eff}}\right)}$ for the static magnetic field *B*_0_ applied parallel to the long axis of the V[TCNE]_*x*_ strip. Here *M*_eff_ = *M*_*s*_ − *H*_*k*_ describes the difference between the saturation magnetization *M*_*s*_ and the uniaxial anisotropy field *H*_*k*_ of the V[TCNE]_*x*_ microstructure.

**Figure 2:**
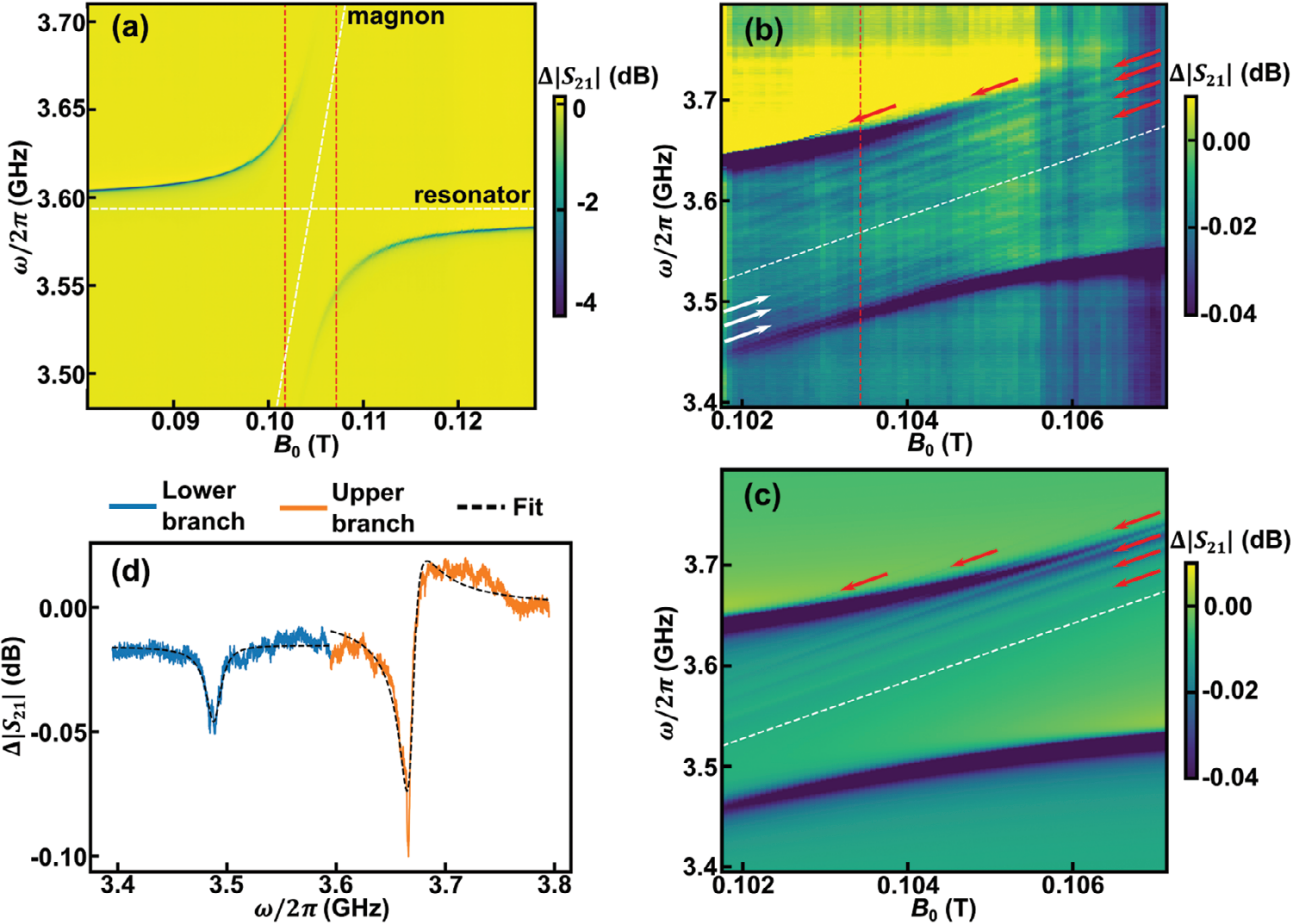
Microwave measurement of V[TCNE]_*x*_ coupled to a 3.6 GHz resonator at 0.43 K. a) Δ|*S*_21_| plotted as a function of magnetic field and frequency. The white dashed lines mark the resonator and the magnon mode frequencies in the case where they are not coupled. b) Δ|*S*_21_| acquired in finer field steps near resonance field (between the red dashed lines in (a)). We attribute the faint diagonal lines with slope 28 GHz/T to be *k* ≠ 0 magnon modes (marked with arrows). Vertical features are artifacts of subtracting non‐magnetic background transmission. c) Simulated transmission spectrum of *k* ≠ 0 magnon modes coupled to the resonator. The model is described in the Supporting Information. d) Δ|*S*_21_| line cut at *B*_0_ = *B*_*res*_ (i.e. Δ = 0, from the red dashed line in Figure 2b.) showing the two branch resonances at ω_+_ and ω_−_. The dashed lines are fits as discussed in the text.

**Figure** **2**a shows the corresponding experimental data Δ|*S*_21_| = 20log_10_(|*S*_21_|/|*S*_21, 0_|) measured at *T* = 0.43 K as a function of magnetic field and frequency, where *S*_21, 0_(ω) is the background transmission. It shows a strong avoided level crossing — direct evidence of strong coupling between resonator photons and magnons. These data are acquired with a VNA using −75 dBm of microwave power at the sample, which is far below the resonator's nonlinear power level, corresponding to about 1.9 × 10^6^ resonator photons for large Δ^[^ ^62^ ^]^ (see the Supporting Information for details). At each field, we extract the resonance frequencies ω_±_ and resonance linewidths κ_±_ of the upper and lower branch by fitting to Equation (2).

To resolve the features at small Δ and to determine *g* and κ_*m*_ accurately, we measure *S*_21_ with finer magnetic field steps. The data are presented in Figure 2b. Qualitatively, the strongest features are the two hybrid photon‐magnon branches dispersing as ω_±_ given by Equation (3). Additionally, we observe fainter modes that are linearly dispersing with a slope of 28 GHz/T. In contrast to the uniform magnon mode that dominates the spectrum, we attribute the modes marked with red arrows to thickness quantized magnon modes with a finite wavevector *k*,^[^ ^35^, ^36^, ^63^, ^64^, ^65^, ^66^ ^]^ which are well described by an input/output theoretical simulation shown in Figure 2c. The modes marked by white arrows are discussed further in the Supporting Information.

Figure 2d shows a line cut of Δ|*S*_21_| as a function of frequency acquired at *B*_0_ = *B*_*res*_ (e.g. Δ = 0), with corresponding fits to Equation (2) for each branch. Because of transmission line standing waves, we fit each mode separately and use the results to determine *g* and κ_±_, which is valid given that κ_±_ ≪ 2*g* ≪ ω_±_. The two resonances appear at the ω_±_ as defined by Equation (3). We note that although ω_−_ has a symmetric resonance lineshape, ω_+_ has a Fano‐like lineshape due to standing waves on the transmission line.^[^ ^61^ ^]^ Nevertheless, we can use the splitting between these resonances to find *g* = [ω_+_(*B*_*res*_) − ω_−_(*B*_*res*_)]/2 = 2π × [90.31(8)] MHz. The damping rates of the two branches, κ_±_ are related to resonator and magnon damping rates via^[^ ^19^ ^]^

(4) $$\begin{array}{c} \kappa_{\pm }=\left[\kappa_{r}/2+\kappa_{m}/2\mp \text{Im}\sqrt{{\left(-\omega_{r}+i\kappa_{r}/2+\omega_{m}-i\kappa_{m}/2\right)}^{2}+4g^{2}}\right] \end{array}$$

From the linewidths in Figure 2d we find κ_−_(*B*_*res*_)/2π = 16.37(29) MHz and κ_+_(*B*_*res*_)/2π = 15.15(17) MHz. At *B*_0_ = *B*_*res*_, the damping rates κ_+_ = κ_−_ = (κ_*r*_ + κ_*m*_)/2. Therefore, we take the average of experimental κ_+_, κ_−_ to estimate (κ_*r*_ + κ_*m*_)/2. Together with κ_*r*_(*B*_*res*_)/2π = 0.902(32) MHz, we estimate κ_*m*_/2π = 30.62(34) MHz. We find that this device operates with C=1181(44) and fulfills *g* > κ_*r*_, κ_*m*_.

Now we return to our analysis of the thickness quantized magnon modes with *k* ≠ 0, highlighting a few key results from our analysis. The coefficients used to produce Figure 2c are listed in **Table** **1**. Although we do not directly fit the spectrum shown in Figure 2b owing to its complexity, we find that to accurately reproduce the Δ|*S*_21_| spectrum from input‐output theory, this choice of parameters is unique up to an overall phase such that the trends and amplitudes that emerge are trustworthy. Additional details are available in the Supporting Information.

**Table 1:** Parameters used to produce Figure 2c.

Thickness quantized magnon modes			
	*g*_*n*_/2π	ω_*mn*_/2π	κ_*mn*_/2π
*n* = 1	30 MHz	ω_*m*0_/2π + 26 MHz	6 MHz
*n* = 2	15 MHz	ω_*m*0_/2π + 46 MHz	5 MHz
*n* = 3	8 MHz	ω_*m*0_/2π + 64 MHz	4 MHz
*n* = 4	3 MHz	ω_*m*0_/2π + 79 MHz	3 MHz
*n* = 5	1.5 MHz	ω_*m*0_/2π + 98 MHz	2 MHz
*n* = 6	1 MHz	ω_*m*0_/2π + 113 MHz	1 MHz

We find that to qualitatively reproduce experimental features, the resonator coupling coefficient, *g*_*n*_ must decrease monotonically with the magnon mode index *n*. This matches the expectation from theory^[^ ^15^ ^]^ (also see discussion in the Supporting Information). In a thickness quantized mode, the net precessing dipole moment of the mode decreases with increasing *n* as different anti‐nodes precess out‐of‐phase with each other. We also notice that with increasing *n*, we must select a magnon mode linewidth that decreases far below the uniform Kittel mode magnon damping rate κ_*m*_ for the modes to be visible. Direct inspection of the data confirms this observation (shown in the Supporting Information). For example, for *n* = 6, we need to use a linewidth of κ_*m*6_ ≈ 1 MHz to reproduce the corresponding feature in the experimental data. This is remarkable because it matches the linewidths observed in YIG spheres and suggests that although planar magnonic devices have some systematic sources of linewidth broadening, the intrinsic linewidth of V[TCNE]_*x*_ microstructures can match those of the best bulk‐grown YIG. To put this in perspective, if we use the *n* = 6 mode to calculate the Gilbert (material) damping α assuming no inhomogeneous broadening or the damping caused by the environment, we get α ≈ 1.4 × 10^−4^, which is competitive with any low‐damping material, even at ambient temperature where two‐level system damping is a small contribution.^[^ ^53^ ^]^ A more complete discussion of linewidth sources and thickness quantized magnon modes, including dispersion and coupling to the resonator, is presented in the Supporting Information.

**Figure 3:**
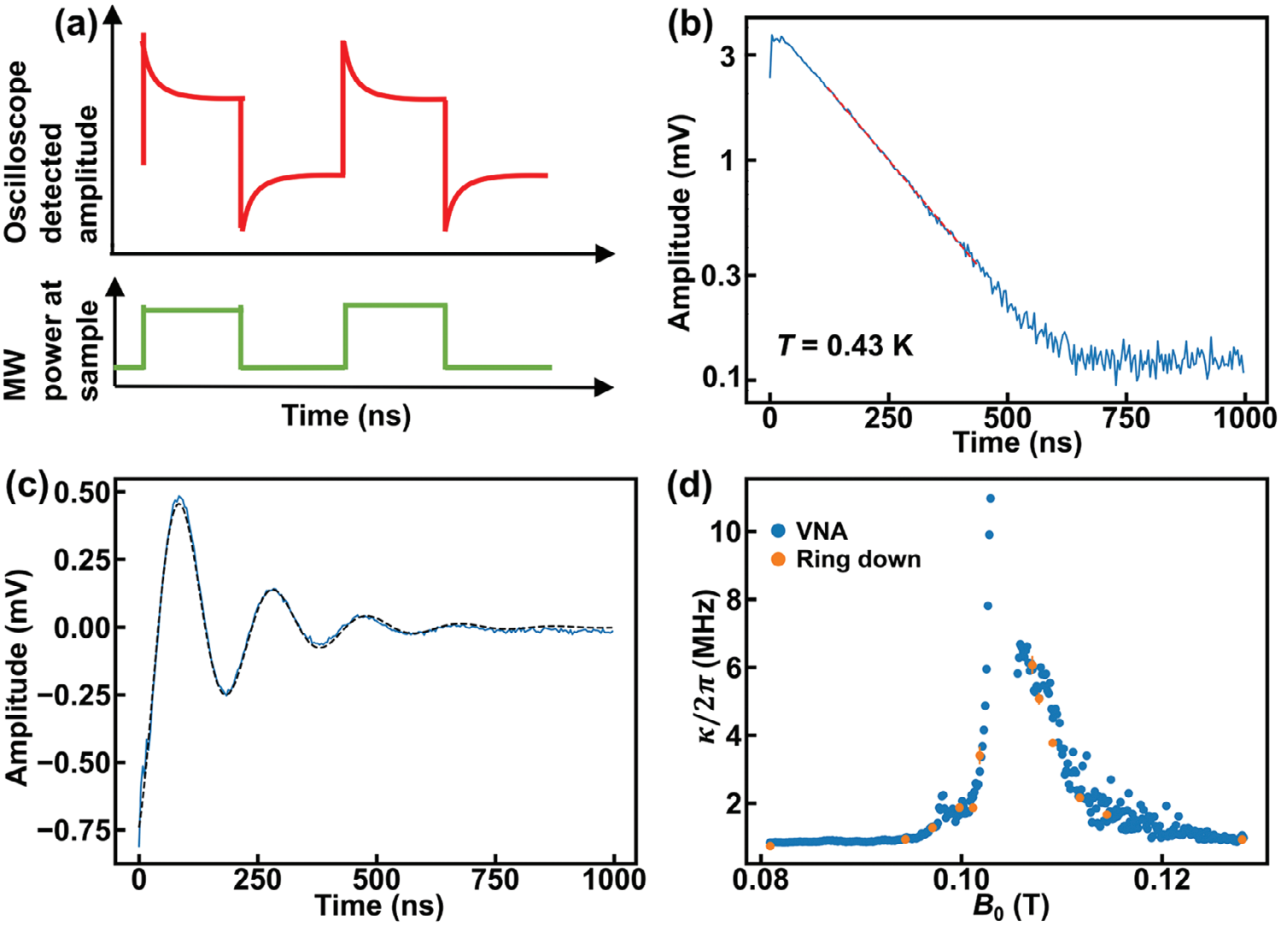
Ring‐down experiment with homodyne circuit for the 3.6 GHz sample at 0.43 K, −65 dBm excitation power. a) Schematic plot of how the homodyne detected signal (red) changes with respect to the microwave excitation power at the sample (green). When the microwave excitation power turns on (off), system will ring up (down) to steady‐state. b) Amplitude versus time plot of ring‐down at 0.101 T for on resonance excitation. The fitted voltage (red dashed line) ring‐down time is 170.0(2.6) ns. c) Amplitude versus time data with 5 MHz detuning (blue) and the fit (black dashed curve). d) Total system damping calculated from ring‐down experiments (orange) and VNA experiments (blue). For most data points, the error bar is smaller than the circular dot.

Having probed the system in the frequency domain with a weak continuous wave drive, we now turn to measurements of system relaxation in the time domain, which probes the system relaxation in the absence of a drive. This relaxation will include both intrinsic relaxation into the environment and relaxation through radiation into the feedline that we detect. The full circuit and measurement protocol is discussed in the Experimental Section. **Figure** **3**a shows the schematic plot of microwave power at the sample and the oscilloscope detected voltage as a function of time.

Figure 3b shows a log‐linear plot of amplitude relaxation after driving the system with −65 dBm of microwave power at the resonance frequency ω_+_ at *T* = 0.43 K and *B*_0_ = 0.101 T. Under these conditions, the hybrid state has more resonator character than magnon character, however, ω_+_ − ω_*r*_ = 2π × 43.9 MHz ≈ *g*/2, indicating substantial magnon participation. We observe exponential relaxation with a voltage ring‐down time constant of 170.0(2.6) ns corresponding to an energy relaxation time constant of 85.0(1.3) ns, and a decay rate of κ_+_/2π = 1.872(29)~MHz. We can also detune the microwave excitation with respect to the hybrid mode. In Figure 3c we plot ring‐down data acquired at the same field, except using a driving frequency that is detuned by 5 MHz from ω_+_/2π. In this case, the homodyne signal beats with the reference oscillator, giving rise to a decaying sinusoidal response. We recover a decay rate of 1.924(28) MHz on top of the 5 MHz beat, which is consistent with the on‐resonance driving result.

To explore the relaxation rates at different values of Δ, we perform on‐resonance ring‐down measurements as a function of *B*_0_. We plot the resulting damping rates along with the damping rates extracted from VNA full‐width at half‐maximum (FWHM) linewidth measurements versus *B*_0_ in Figure 3d. We see excellent agreement between the two methods. Figure 3d underscores that the magnon damping rate is one order of magnitude larger than the bare resonator damping rate, evident by an increase in κ_±_ as |Δ| approaches 0. The upper branch damping rate at 0.0809 T and the lower branch damping rate at 0.1281 T are assumed to be the resonator damping rates with negligible magnon contribution (see the Supporting Information for more details). By linear interpolation, we estimate κ_*r*_/2π to be 0.902(32) MHz at *B*_*res*_, which we used above to calculate C.

Our experimental results and theoretical analysis highlight the opportunities for low‐damping cavity magnonic systems and planar quantum circuit integration using V[TCNE]_*x*_  and related materials, and reveal a new path toward ultra‐low magnon damping in planar systems. The fact that magnon modes with *k* ≠ 0 have much smaller linewidths than the uniform magnon mode provides interesting design opportunities. For example, although such modes naturally couple more weakly to the resonator due to their smaller net dipole moment, strategies that incorporate deliberate design for coupling to finite *k* modes could strengthen the coupling. For example, introducing spatial modulation in the resonator inductor wire with periodicity that matches the *k* on in‐plane magnon modes or introducing spatial modulation in the magnetic material itself are each interesting directions to explore. Each of these are experimentally reasonable in V[TCNE]_*x*_, with magnon wavelengths in the range of 100's of nm.

Another important question for achieving ultra‐low magnon damping is understanding the role of two‐level system (TLS) coupling to a magnon mode of interest. Our experiment probes the κ_*m*_ of V[TCNE]_*x*_ modes at a few temperatures between 0.43 and 3 K (see the Supporting Information), where we observe only weak changes to κ_*m*_ as a function of temperature, likely consistent with TLS interactions (see the Supporting Information). Our observation is consistent with a recent study of V[TCNE]_*x*_ ferromagnetic resonance linewidth as a function of temperature between 5  and 300 K in which the TLS contribution was also found to be weak,^[^ ^53^ ^]^ which is promising for integrated quantum applications because it suggests that V[TCNE]_*x*_  is insensitive to intrinsic TLS impurities. Future work extending these findings to *T* ≲ 50 mK could provide additional insight into the TLS physics of V[TCNE]_*x*_.

## 3 Conclusion

In conclusion, we have demonstrated a cavity magnonic system composed of superconducting resonator photons and magnons in the strong coupling regime with C exceeding 1000. A key advance of this work is the integration of lithographically patterned and low‐damping magnetic material with a superconducting circuit platform, contributing to new quantum technologies in which the electrical and the magnonic structures are designed and fabricated on an equal basis. This capability can lead to low‐loss and scalable magnonic quantum circuit elements, new forms of hybrid‐system couplers, new opportunities for tunability, and the creation of transmon qubits with integrated magnonic properties. Beyond its potential for future quantum technologies, high‐cooperativity V[TCNE]_*x*_‐based cavity magnonic systems can offer sensitive readout of coherent magnonic excitations. The broad design space offered by lithographic patterning allows experiments that selectively probe different magnon wavevectors and – in combination with superconducting qubits – enables the creation, manipulation, and detection of single magnons in a scalable, integrated platform.

## 4 Experimental Section

### Ring Down Experiment

For the ring down experiment, pulsed microwave excitation was applied to the feedline and detect the ring‐up/‐down response in the time domain using a homodyne circuit. The microwave pulses were long enough to excite the system into driven steady‐state oscillations. When the microwave drive turned off suddenly, the system would continue to oscillate, however, it did so at its natural frequency. The signal was amplified and mixed with the reference tone, and the result was digitized.

The setup for the ring‐down experiments is shown in Figure S13 (Supporting Information). From the top‐left of this diagram: the microwave (MW) signal generator generated a sinusoidal voltage at a frequency relevant to ω_+_ or ω_−_, which was then split using a directional coupler. The top branch, which labeled as the local oscillator, carries 11 dBm of MW power. The lower branch, which labeled as the signal carrier, carries −9 dBm of MW power. In the lower branch, a function generator output a square wave that controls a microwave switch that chops the lower branch microwave power in a square wave. After the switch, the attenuation from the coaxial cable, a 20 dB attenuator outside the cryostat, and a 30 dB attenuator inside the cryostat attenuate the MW signal to −65 dBm before it reached the sample. The signal from the sample was amplified outside the cryostat before it was mixed with the local oscillator signal, with a power limiter before the mixer to protect it from overloading. The phase shifter was adjusted in the local oscillator branch to maximize the DC component of the mixed ring‐down signal. At the end, a low pass filter rejected components at 2ω and A DC component of the mixed signal was left that it was digitize using a sampling oscilloscope. The resulting voltage was proportional to the amplitude of magnon‐resonator ring‐down, which was fitted as discussed in the main text.

### Fabrication and Magnet‐Resonator Integration

The fabrication procedure must integrate the growth and patterning of inorganic superconducting films with organic‐based V[TCNE]_*x*_.^[^ ^42^, ^54^ ^]^ This began by sputtering 60 nm of Nb (critical temperature *T*_*c*_ = 7.3 K) on a sapphire wafer and patterning the Nb film using photolithography followed by dry etching. First, the wafer was spin coated with photoresist, exposed using a 5X g‐line stepper, and then developed. Then the Nb was etched using ion milling before stripping the photoresist.

In preparation for V[TCNE]_*x*_ deposition, a poly‐methyl methacrylate (PMMA) poly(methyl methacrylate‐methacrylic acid) (P(MMA‐MAA)) bilayer on an individual die was spin coated, and it was exposed using electron beam lithography. Once baked and developed, ≃3 nm of Al was evaporated to suppress diffusion of any residual volatiles in the polymer film. The resulting structure was a lift‐off template for CVD‐deposited V[TCNE]_*x*_.^[^ ^41^ ^]^ The die was transferred into an argon‐purged glovebox, wherein the V[TCNE]_*x*_ was deposited at ≈ 60 °C^[^ ^47^, ^48^, ^55^ ^]^ and then lift‐off was performed by dissolving the polymer film using dichloromethane. The V[TCNE]_*x*_ was encapsulated by epoxy and a glass coverslip while still in the glovebox, stabilizing the material for weeks under ambient conditions.^[^ ^42^, ^54^ ^]^ Ossila E131 epoxy (developed for organic light emitting diode displays) was used. It was drop cast on the V[TCNE]_*x*_ strip. Then a smaller glass coverslip was placed on top. The cover slip and epoxy only cover half of the device to allow for wire‐bonding. The ensemble was cured under white light for 1 h. Figure 1a shows an optical micrograph of an encapsulated device with a resonator frequency ω_*r*_/2π ≈ 3.6 GHz. A zoom into the narrow inductor shows the bright 10‐µm‐wide Nb wire with a dark gray strip of patterned V[TCNE]_*x*_ running down its center. The V[TCNE]_*x*_ strip was 300 nm thick, 6 µm wide and 600 µm long.

### Magnetic Field Calibration

To calibrate the magnetic field of the cryostat electromagnet at the sample location, absorption electron spin resonance (ESR) of 2,2‐diphenyl‐1‐picrylhydrazyl (DPPH) was measured in contact with the central conductor of a broadband coplanar waveguide. DPPH was a stable radical with a well‐studied gyromagnetic ratio across a broad range of temperatures. The ESR was measured at 4.7 K, where the *g* factor was 2.083.^[^ ^67^ ^]^ ESR was measured from 4.5  to 8.5 GHz in steps of 0.5 GHz. At each frequency the magnet current was swept while acquiring microwave transmission with a vector network analyzer. The magnet current associated was extracted with the spin resonance at each frequency. It was found that the line formed by plotting the *B*_0_ (calculated from frequency divided by the gyromagnetic ratio of DPPH) as a function of resonant currents extrapolates to the origin as expected. Using these data a calibration factor of 60.64(10) mT/A was calculated with an uncertainty in absolute magnetic field of 0.4 mT. Figure S14 (Supporting Information) shows a plot of the static field at the sample *B*_0_ versus the magnet coil current.

### Statistical Analysis

Uncertainty was calculated via standard error analysis from least‐squared fitting. The python package “scipy.optimize.curve_fit”was used.^[^ ^68^ ^]^ The output included the optimized values of all the fitting parameters and a 2‐D array “pcov” which gave the covarience matrix. The square root of the diagonal entries of the covariance matrix as the standard error was reported.

## Conflict of Interest

The authors declare no conflict of interest.

## Author Contributions

G.D.F, E.J‐H., and M.E.F. conceived of the experiment. Q.X., H.F.H.C., D.S.C, S.P., H.Y., M.C., E. J‐H., and G.D.F. developed the fabrication procures, designed the devices, and fabricated the devices. D.S.C. and H.Y. optimized V[TCNE]_*x*_ deposition. Q.X., H.F.H.C., D.S.C., T.O.P., M.E.F., E.J‐H., and G.D.F. analyzed the data and developed the theoretical analysis. The paper was written by all authors.

## Supporting information

Supporting Information

## Data Availability

The data that support the findings of this study are available from the corresponding author upon reasonable request.
