# Supplementary material for: Strong Photon‐Magnon Coupling Using a Lithographically Defined Organic Ferrimagnet
Source: Adv Sci (Weinh). 2024 Jan 26;11(14):2310032. doi: 10.1002/advs.202310032 (PMC11005739; doi:10.1002/advs.202310032)
Supplement: Supplementary file 1 — Supporting Information [file ADVS-11-2310032-s001.pdf]

ADVANCED SCIENCE
Open Access

Supporting Information

for *Adv. Sci.*, DOI 10.1002/advs.202310032

Strong Photon-Magnon Coupling Using a Lithographically Defined Organic Ferrimagnet

*Qin Xu, Hil Fung Harry Cheung, Donley S. Cormode, Tharnier O. Puel, Srishti Pal, Huma Yusuf, Michael Chilcote, Michael E. Flatté, Ezekiel Johnston-Halperin and Gregory D. Fuchs**

Supplementary Appendix: Strong photon-magnon coupling using a lithographically defined organic ferrimagnet

Qin Xu,[1] Hil Fung Harry Cheung,[1] Donley S. Cormode,[2] Tharnier O. Puel,[3] Srishti Pal,[4] Huma Yusuf,[2] Michael Chilcote,[4] Michael E. Flatté,[3] Ezekiel Johnston-Halperin,[2] and Gregory D. Fuchs[4]

[1]*Department of Physics, Cornell University, Ithaca NY 14853*

[2]*Department of Physics, The Ohio State University, Columbus, OH 43210*

[3]*Department of Physics and Astronomy, University of Iowa, Iowa City, IA 52242*

[4]*School of Applied and Engineering Physics, Cornell University, Ithaca NY 14853*

(Dated: December 8, 2023)

I. ESTIMATED COUPLING AND DESIGN

One can estimate the single spin coupling rate from the geometry as [1, 2] $g_s = g_e\mu_B b_{rf}\omega_r/\sqrt{8\hbar Z_r}$, where $b_{rf} = \mu_0/2w$ is the magnitude of the magnetic field experienced by V[TCNE]$_x$ spins per unit current in an inductor wire of width w when the spins are in close contact. We have also used the electron Landé g factor g_e, the Bohr magneton μ_B, and the characteristic impedance of the resonator $Z_r = \sqrt{L/C}$.

Using our estimated device's characteristic impedance Z_r=17.0(4.5) Ω and inductor width w = 10 μm, we estimate $g_s/2\pi$ = 36(5) Hz. Using $N = 2.195 \times 10^{12}$ (from the magnetic volume described below, and M_s), the total coupling rate is estimated to be $g/2\pi$ = 54(8) MHz. The resonator is capacitively coupled to a coplanar microwave feedline that we use to excite and detect the coupled resonator-magnon system.

One of the unusual properties of V[TCNE]$_x$ as a magnetic material is that it has a relatively low value of the saturation magnetization $\mu_0 M_s \sim 10$ mT [3–5]. On one hand, this could be a disadvantage in reaching a large $\sqrt{N}$ to enable strong coupling. On the other hand, it is advantageous from a device design point of view because it allows one to work at comparatively small applied magnetic field, which avoids superconducting vortex formation.

II. DEVICE SIMULATIONS

We simulate the resonator with Keysight PathWave Advanced Design System (ADS) software. Fig. S1(a) shows the simulated transmission coefficient $\Delta|S_{21}|$ vs frequency for the 3.6 GHz resonator. The simulated resonance frequency is 3.933 GHz. We then simulate the device being driven at the resonance frequency and plot the time averaged magnitude of current density in the superconducting film. The result is shown in Fig. S1(b), in which red indicates larger current density and blue indicates smaller current density. This resonance mode has a large current density in the inductor wire for efficient coupling to magnon modes of a magnetic material deposited on the wire. After fabrication, we measure the resonator's transmission spectrum using a VNA at 0 field before V[TCNE]$_x$ deposition. The result is shown in Fig. S1(c), where the resonance is at 3.804 GHz with Q-factor (Q_l) of 4922. This resonance frequency is lower than the simulation because of the finite kinetic inductance of Nb [6, 7], which is not included in the simulation. This kinetic inductance adds to the

geometric inductance of the LC resonator and decreases the resonance frequency. Fig. S1(d) shows the transmission spectrum at 0 field after V[TCNE]$_x$ deposition and encapsulation. The encapsulation epoxy and cover glass increase the effective dielectric constant of the resonator environment, which increases the capacitance C, resulting in a lower resonance frequency. We speculate that the decrease of Q (2546) with epoxy encapsulation is caused by the loss tangent of the epoxy and glass, and loss from the non-uniform V[TCNE]$_x$ magnetization at 0 field. A non-uniform V[TCNE]$_x$ magnetization means some domains are not aligned, which will cause a spatially varying fringe B field that can penetrate the superconducting inductor wire and cause vortices. Vorticies introduce an additional loss mechanism that will decrease Q. Then we increase the field to 0.0809 T to saturate V[TCNE]$_x$ magnetization. Fig. 1(b) in the main text shows the resulting transmission spectrum. We attribute the increase in Q (4302) to the increase of the uniformity of the V[TCNE]$_x$ magnetization.

III. RESONATOR LOSS FROM EPOXY AND COVER GLASS

The epoxy and cover glass decrease the resonator's internal quality factor Q_i defined by [8] $Q_i^{-1} = Q_l^{-1} - \mathrm{Re}(Q_c^{-1})$. We measure a bare resonator of the same design to have $Q_i = 13606$. However, for a resonator encapsulated with epoxy and glass slide without V[TCNE]$_x$, Q_i decreases to 9070. We note that other, lower-loss encapsulation strategies are being pursued, and that this is not a fundamental limitation.

IV. RESONATOR AND MAGNON MODE SATURATION POWER

Using power-dependent S_{21} measurements at $B_0 = 0.0994$ T, we determine the saturation power (of the kinetic inductance non-linearity) for the 3.6 GHz resonator to be larger than -65 dBm applied to the feedline port. To make sure we are not saturating the magnetic resonance, we also make power-dependent S_{21} measurements near B_{res}. No significant power dependence is observed in the range of -65 dBm to -85 dBm (Fig. S9(b)). The data in Fig. 2 are acquired with -75 dBm of microwave power at the sample, which is well below saturation for both the resonator and the magnet.

We now theoretically estimate the magnon precession cone angle. When we drive on resonance with the upper or lower branch ω_{res}, the average number of excitations in the

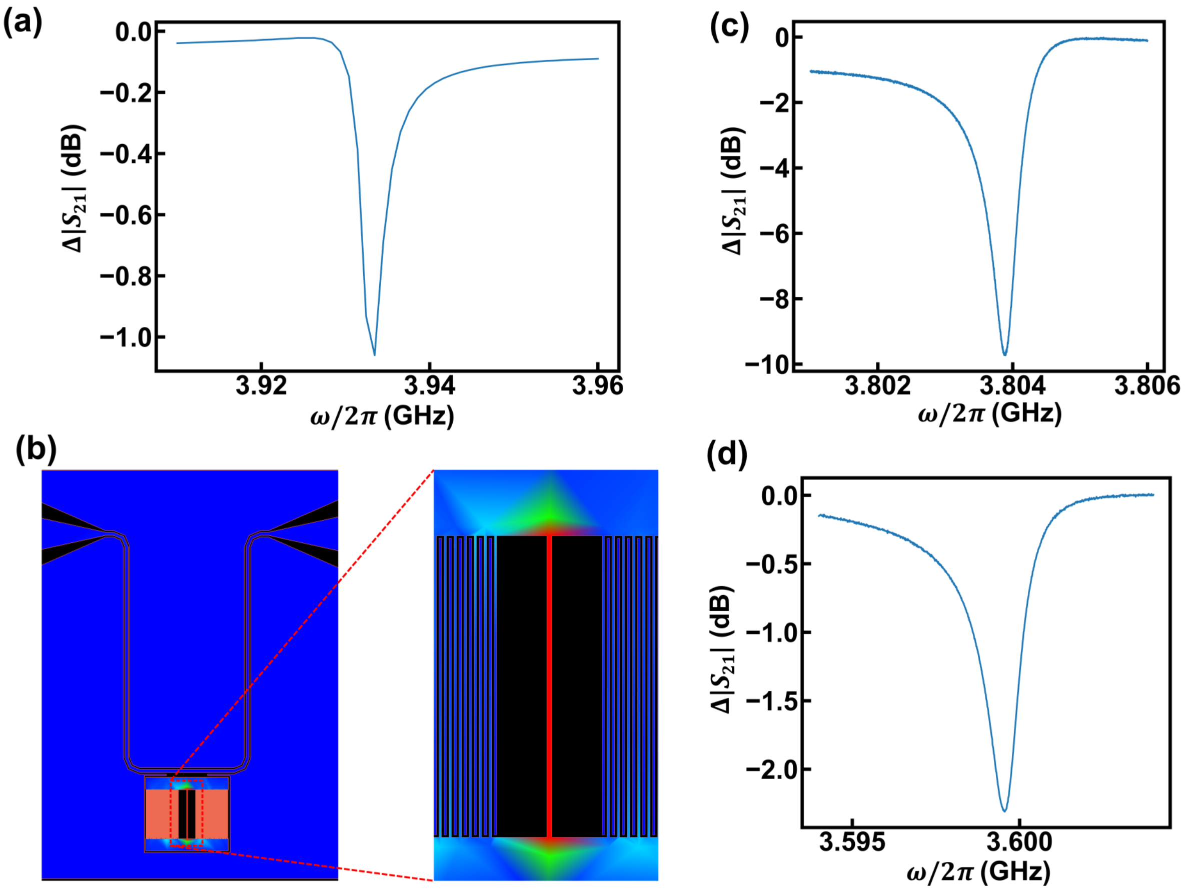

FIG. S1. (a) Microwave transmission $\Delta|S_{21}|$ from the ADS simulation of the 3.6 GHz resonator. We find a resonance frequency of 3.93 GHz. (b) The time averaged magnitude of current density plot of the resonator at the resonance frequency shown in (a). The zoom shows a large current density in the inductor wire. (c) Experimental measurement of $\Delta|S_{21}|$ at $B_0 = 0$ for a resonator at 0.43 K without V[TCNE]$_x$. Fitting with equation (2) gives $\omega_r/2\pi = 3.804$ GHz and $Q_l = 4922$. The resonance frequency is lower than the simulation because of the finite kinetic inductance of Nb in the real device, which is not included in the simulation. (d) Experimental measurement of $\Delta|S_{21}|$ at $B_0 = 0$ for the V[TCNE]$_x$-resonator device discussed in the main text, at 0.43 K. Fitting with equation (2) gives $\omega_r/2\pi = 3.600$ GHz and $Q_l = 2546$.

resonator-magnet system is [9]

$$\langle n \rangle = \frac{2P_{in}}{\hbar\omega_{res}^2}\frac{Q_l^2}{|Q_c|},$$

where $P_{in} = -75$ dBm $= 3.16 \times 10^{-11}$ W is the excitation power at the sample, Q_l is the

loaded Q-factor and Q_c is the coupling Q-factor. For the upper branch at $B_0 = B_{res}$, we find $Q_l = 242.1$, $|Q_c| = 22810$ and the resonance frequency $\omega_{res}/2\pi = 3.669$ GHz from fitting to equation (2). Under these conditions, we estimate $\langle n \rangle = 2900$ and the average number of magnons $\langle n_m \rangle = \langle n \rangle / 2 = 1450$ at $B_0 = B_{res}$.

The cone angle θ of the uniform magnon mode satisfies

$$1 - \cos\theta \approx \frac{1}{2}\theta^2 = \frac{\langle n_m \rangle \hbar \omega_{res}}{\frac{1}{2} N \hbar \omega_{res}},$$

where $N \approx 2.195 \times 10^{12}$ is the estimated number of V[TCNE]$_x$ spins in the sample. Therefore, we estimate $\theta \approx 2\sqrt{\langle n_m \rangle / N} = 5.1 \times 10^{-5}$ rad $= 0.0029°$.

Similarly, in Fig. 1(b) with $\omega_{res}/2\pi = 3.604$ GHz, $Q_l = 4302$ and $|Q_c| = 11200$, we get the average number of resonator photons $\langle n_r \rangle \simeq \langle n \rangle = 1.9 \times 10^6$.

V. EXTRACTED RESONATOR AND MAGNETIC PARAMETERS

We find that ω_r and κ_r have weak but non-zero magnetic field dependence. We attribute this behavior to vortices in the superconducting film that can decrease ω_r and increase κ_r [6, 7]. Therefore, to characterize the resonator independently of magnon hybridization, we wish to measure it at a field with a large detuning from the degeneracy point with the magnon modes. However, if we detune too much, the frequency and linewidth is not correct. Additionally, at zero field, the linewidth is larger due to vorticies created by the nonuniform magnetization of the V[TCNE]$_x$. Therefore, we seek to characterize the resonator at a magnetic fields that have large detuning Δ relative to g. Additionally, at very high field there are more vorticies from the small out-of-plane component of magnetic field. We choose two optimal fields, $B = 0.0809$ T and $B = 0.1281$ T, which correspond $\Delta = -7.2g$ (1.89% of Kittel mode participation) and $7.8g$ (1.59% of Kittel mode participation), respectively. Using the measurements at this field, we model these contributions phenomenologically, assuming that they vary linearly with magnetic field.

Using the data shown in Fig. 2(a), we fit $\omega_\pm$ by fitting to equation (2). These results are then fit to equation (3) where ω_m is given by the Kittel formula with $\gamma/2\pi = 28$ GHz/T. Also, we assume $\omega_r = \omega_{r0} + \gamma_r B_0$ and treat ω_{r0} and γ_r together with M_{eff} and g as free parameters. We have used $M_{\text{eff}} = M_s - H_k$, where H_k is the uniaxial anisotropy field. We obtain $\omega_r = 3.593$ GHz at $\Delta = 0$ and $\mu_0 M_{\text{eff}} = 53.614(63)$ mT. Such a large M_{eff} is likely

caused by the large strain applied to the V[TCNE]$_x$ due to differential thermal expansion [5], which can induce a value of $H_k > M_s$.

Using these two measurements, we calculate the average resonator linewidth to be 0.902(32) MHz, which we take to be an upper bound on κ_r Additionally, we establish the resonator frequency at $\Delta = 0$

Next we determine the value of B_{res} using the data shown in Fig. 2(b), again extracting $\omega_\pm$ by fitting to equation (2). Fig. S2 shows the extracted splitting $\omega_+ - \omega_-$ as a function of B_0. From equation (3) we know that $\omega_+ - \omega_- = \sqrt{\Delta^2 + 4g^2}$, where $\Delta = \omega_m - \omega_r = \gamma_{rm} \times (B_0 - B_{res})$, and γ_{rm} is the change of $\omega_m - \omega_r$ per unit increase of B_0. Treating g, B_{res} and γ_{rm} as free parameters, we get $g/2\pi = 90.43(8)$ MHz, $B_{res} = 0.103429(18)$ T and $\gamma_{rm}/2\pi = 52.1(2.6)$ GHz/T. The best-fit curve is shown as the black dashed line in Fig. S2. First, we find a value of g that is consistent with the value we extracted from the line cut shown in Fig. 2(d). However, the fitted $\gamma_{rm}/2\pi$ is larger than the expected value of 28 GHz/T. Possible reasons for the discrepancy include (1) that interactions between the $k \neq 0$ magnon modes and the upper (lower) branch distorts the shape of $\omega_+(B_0 > B_{res})$ and $\omega_-(B_0 < B_{res})$ from the model prediction of equation (3) (see Fig. S3); (2) Although the fit requires a γ_{rm}, the resonances that we use to extract it are strongest for larger values of Δ, where its influence is small. Thus, we are not as sensitive to γ_{rm} than we are to other parameters, and small covariances and distortions can lead to an unphysical value. Nevertheless, the most relevant parameters for this analysis, g and B_{res} are insensitive to the exact value of γ_{rm}.

VI. MAGNON MODES

As discussed in the main text, we find additional magnon modes that are evident in Fig. 2(b), i.e., the lines indicated by arrows within the avoided level crossing region. For reference, we plot a dashed line showing the uniform magnon mode position as if it were not coupled to the resonator. It separates the additional modes into higher frequency (red arrows) and lower (white arrows) frequency modes, which likely have distinct origins. Here, we treat all magnon modes as spinwave modes characterized by a wavevector $\boldsymbol{k}$ and quantized by the boundary conditions at the surfaces of the V[TCNE]$_x$. The case $k = 0$ refers to the uniform magnon mode, while the additional modes are $k \neq 0$ magnon modes. The width

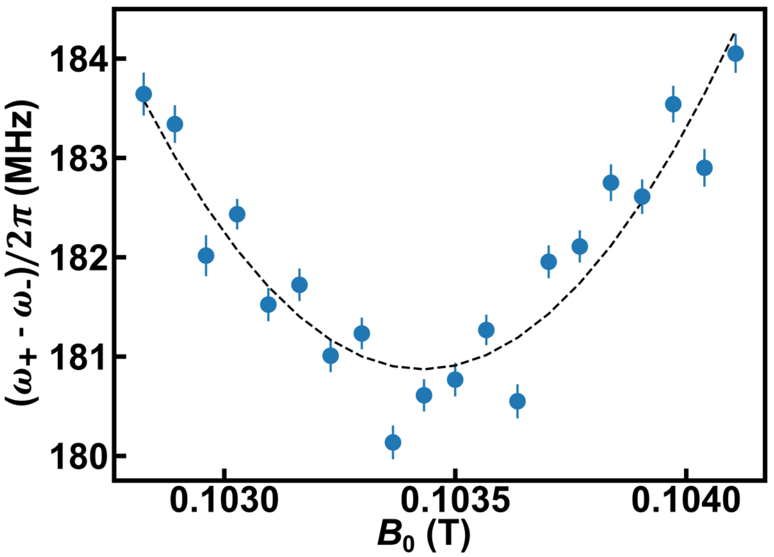

FIG. S2. Upper and lower branch frequency difference vs field and the fitting to extract g and B_{res}. At the resonance field, the frequency difference is the smallest and is equal to $2g$.

and length of the V[TCNE]$_x$ strip are long enough that quantization constrained in those directions cannot be resolved; only modes that are quantized in the thickness direction ($|\boldsymbol{k}| \equiv k$ perpendicular to the magnetic field) are resolvable.

To better understand the transmission spectrum, we now discuss a toy model for multiple magnon modes coupled to the resonator. Later we discuss deviations between the experimental observations and the toy model. First we extend the Hamiltonian $\mathcal{H}_0$ given in equation (1) by adding a term $\mathcal{H}_s$ that describes the $k \neq 0$ magnon modes as

$$\mathcal{H}_s/\hbar = \sum_{n=1} \omega_n \hat{b}_n^\dagger \hat{b}_n + \left(\sum_{n=1} g_n (\hat{b}_n^\dagger \hat{a} + \hat{b}_n \hat{a}^\dagger) \right). \tag{S1}$$

We introduce creation and annihilation operators $\hat{b}_n^\dagger$ and $\hat{b}_n$ for the $k \neq 0$ magnons, and their direct coupling to the resonator has strength g_n.

We assign the features at frequencies above the dashed line to be thickness quantized $k \neq 0$ magnon modes, which are modes described by dipole-exchange interactions [10]; their frequency dispersion is given in Ref. [11],

$$\omega_n = \gamma \sqrt{(B_0 + \mu_0 M_{\rm eff} \lambda_{\rm ex}^2 k_n^2)\,(B_0 + \mu_0 M_{\rm eff} + \mu_0 M_{\rm eff} \lambda_{\rm ex}^2 k_n^2)}. \tag{S2}$$

The quantization index $n = 1, 2, \ldots$ is along the thickness direction, where the wavevectors are constrained by $k_n L = n\pi$. Setting $n = 0$ recovers the frequency of the uniform magnon

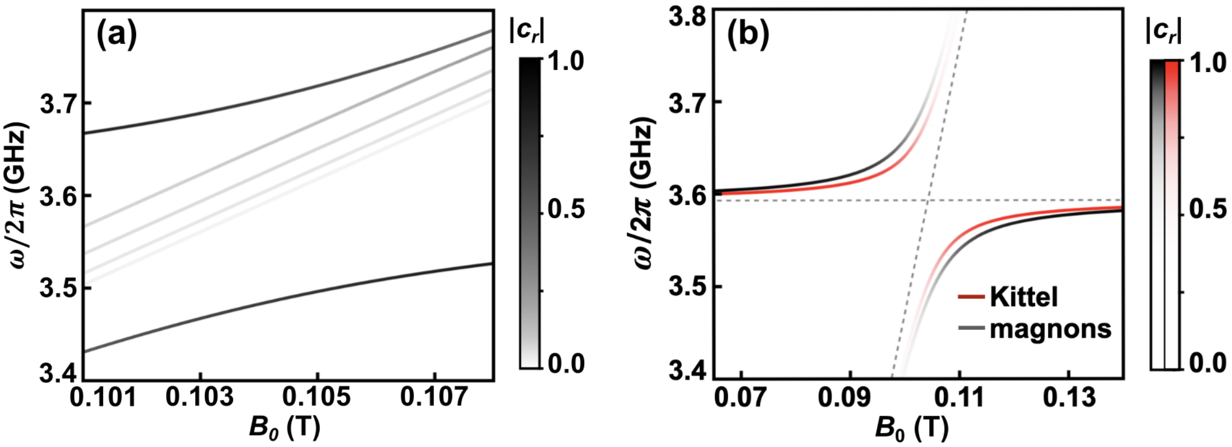

FIG. S3. (a) Theoretical energy spectrum of $k \neq 0$ magnon modes coupled to the resonator. (b) The results labeled by 'magnons' repeat (a) , while the red lines include only the coupling between the uniform magnon mode (Kittel mode) and the resonator mode (both independently identified by the dashed lines). This simulation used the following realistic parameters: L = 300 nm, $\gamma/2\pi = 28$ GHz/T, $\mu_0 M_{\text{eff}} = 53.6$ mT, $\lambda_{\text{ex}}^2 = 0.25 \times 10^{-16}$ m^2, $\omega_r/2\pi = 3.593$ GHz, $g/2\pi = 90$ MHz, and $g_n = g/(n+1)$ for $n = 1, \ldots, 4$ (likely to be an overestimate, to increase the contrast in the figure).

mode (or Kittel mode), while the extra terms are due to the presence of exchange interactions with amplitude described by the exchange-length constant λ_{ex}. The expression above assumes B_0 is oriented in the plane of V[TCNE]$_x$.

These higher frequency magnon modes can be directly excited by the magnetic field generated by the inductor only if their spatially-averaged amplitude does not vanish; therefore, their coupling to the resonator is highly dependent on the spin-pinning boundary conditions [4, 12]. For instance, there is no direct coupling for totally unpinned boundaries, while for complete pinning only odd n-index modes couple. Here, we consider an intermediate situation where all g_n are allowed to exist, which can happen for partial pinning [13]. To demonstrate the essential features of this interaction, we chose $g_n = g/(n+1)$ as a qualitative description of the features observed, which is likely an overestimate. We solve the equation $\mathcal{H}\,|\psi_i\rangle = \hbar\omega_i\,|\psi_i\rangle$ as a function of B_0 and plot the results in Fig. S3(a). The two branches $\omega_\pm$ represent the hybridized modes due to the strong uniform magnon-resonator coupling. The $k \neq 0$ modes have weak $\Delta|S_{21}|$ (Fig. S3(a)) because the $|\psi_i\rangle$ have small resonator amplitudes, however, they become stronger as their frequencies approach the ω_+

branch (Fig. S3(a)). We truncate the plot at $n = 4$ capturing only magnon modes that lie within the avoided-crossing gap, however, we note that there are higher n modes present in the experiment, which are discussed further below. The spacing between the $k \neq 0$ magnon modes is sensitive to the parameter λ_{ex} through a linear dependence on λ_{ex}^2. The coupling of the $k \neq 0$ magnons with the resonator increases the total gap between the two branches $\omega_\pm$, as shown in Fig. S3(b).

For direct comparison with experimental data, it is useful to introduce the input-output formalism to directly calculate $\Delta|S_{21}|$ from resonance parameters including the superconducting resonator and all magnon modes. Although the data shown in main text Fig. 2(b) is too complicated to fit directly, we can extract parameters from the data and use calculations of $\Delta|S_{21}|$ to compare with the experiment. We can also test hypotheses about resonance parameters to be sure they are consistent with our picture of the physics of the sample. We adapt the formula from Ref. [14] , including the quantized thickness magnon modes along with the uniform mode:

$$\Delta|S_{21}|(\omega, B_0) = \left| 1 + \frac{(\kappa_{ext}/2)e^{-i\phi}}{i(\omega - \omega_r) - \kappa_r/2 + \frac{g_0^2}{i(\omega - \omega_{m0}(B_0)) - \kappa_{m0}/2} + \sum_{n=1}^{\infty} \frac{g_n^2}{i(\omega - \omega_{mn}(B_0)) - \kappa_{mn}/2}} \right|.$$

To generate Fig.2(c) of the main text, we manually choose these parameters to most closely match Fig. 2(b). The parameters and the simulated plot are shown in Table I and Fig. S4(b), where larger values of n correspond to higher frequency magnon modes.

There are several observations about these chosen parameters. Firstly, to reproduce Fig. 2(b), we must choose a decreasing g_n as n increases, consistent with the theory discussed above. If we do not decrease g_n consistent with the theory, the predicted $\Delta|S_{21}|$ are qualitatively different from the experimental data. Secondly, we find that $\kappa_{mn} < \kappa_{m0}$ for all $n \geq 0$. This can be seen directly from linecuts of the data shown in Fig. S4(c) and (d). For higher n magnon modes, the smaller linewidth/decay rate is similar to the magnon damping rates observed in YIG spheres, which have a Gilbert damping similar to that of V[TCNE]$_x$. We speculate that because the higher n modes have a smaller net precessing dipole moment, owing to every other antinode precessing out of phase and thus leading to internal field cancellation, the larger damping rate of the uniform mode magnon may be due to coupling to lossy electromagnetic modes besides the LC resonator.

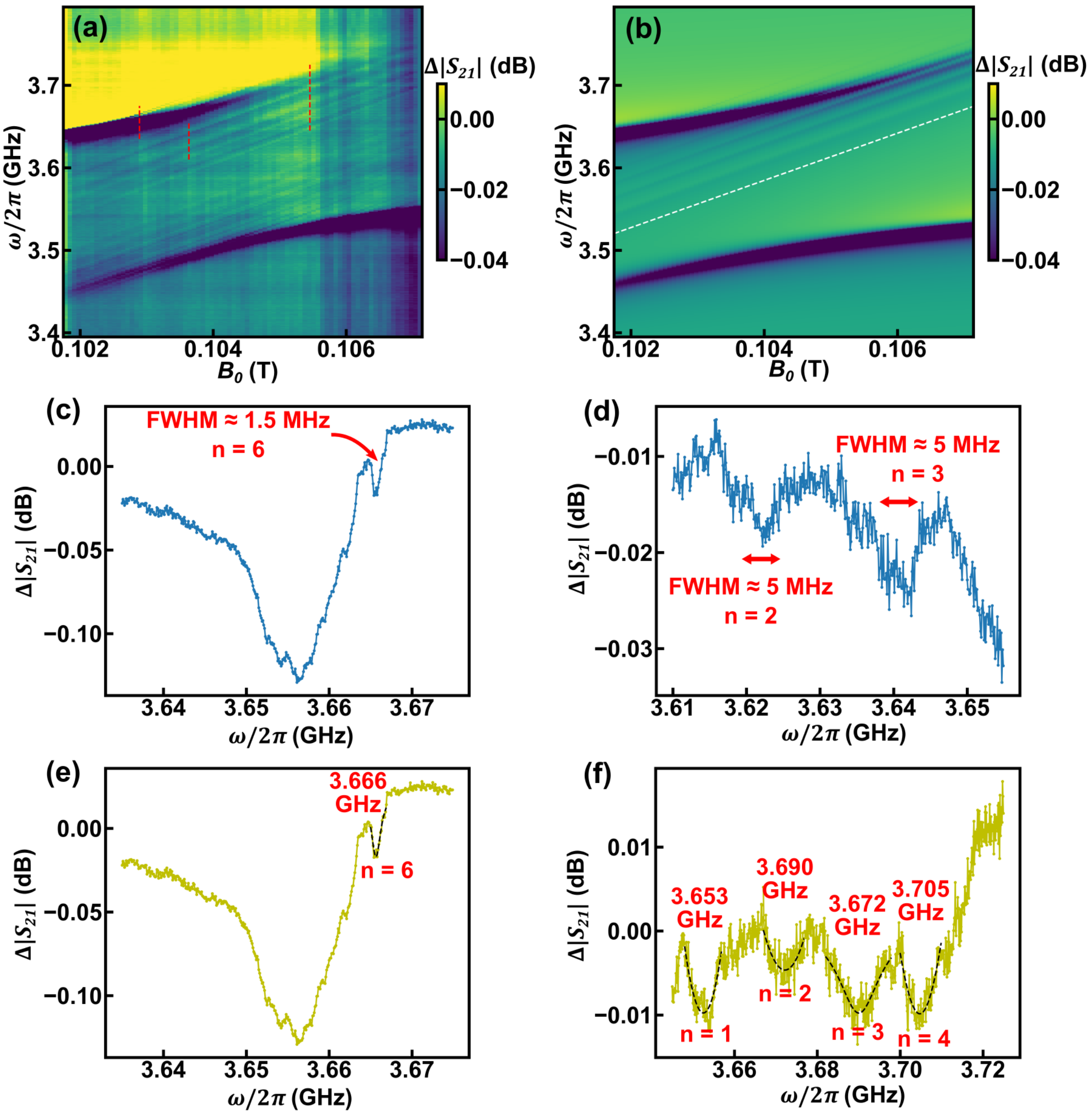
(a)
(b)
$\Delta|S_{21}|$ (dB)
$\omega/2\pi$ (GHz)
B_0 (T)
(c)
FWHM ≈ 1.5 MHz
n = 6
(d)
FWHM ≈ 5 MHz
n = 3
FWHM ≈ 5 MHz
n = 2
(e)
3.666 GHz
n = 6
(f)
3.653 GHz
3.690 GHz
3.672 GHz
3.705 GHz
n = 1
n = 2
n = 3
n = 4

FIG. S4. (a) Experimental $\Delta|S_{21}|$ data as a function of magnetic field and frequency at 0.43 K (same as Fig. 2(b)). (b) Simulated $\Delta|S_{21}|$ as a function of magnetic field and frequency (Same as Fig. 2(c)). (c) $\Delta|S_{21}|$ line cut at $B_0 = 0.10289$ T (the left red dashed line in (a).) showing signals related to the $n = 6$ magnon resonance. The estimated FWHM of this dip is labeled. (d) $\Delta|S_{21}|$ line cut at $B_0 = 0.10363$ T (the middle red dashed line in (a).) showing signals related to $n = 2$ and 3 magnon modes. The estimated FWHM's of these two dips are labeled. (e) and (f) shows a linecut at $B_0 = 0.10289$ T and 0.10546 T (the left and right red dashed lines in (a)) to extract the resonance frequencies of the magnon mode signals for n = 1, 2, 3, 4 and 6. From these fitted frequencies we obtain the values of ω_{mn} shown in Table I. The signal for n = 5 magnon mode is too weak for us to fit, so we manually adjust the simulated frequency to qualitatively reproduce the result near $B_0 = 0.1043$ T and $\omega/2\pi = 3.690$ GHz. Note that the FWHM's in (c) and (d) are larger than κ_{m2}, κ_{m3} and κ_{m6} that used to produce (b) because these dips are hybrid modes with some higher damping inherited by participation by the uniform mode.

A third observation revealed by Table I is that the magnon modes are roughly evenly spaced in frequency, which doesn't match the prediction of the toy model of thickness quantized modes discussed above. In the toy model we assumed that the V[TCNE]$_x$ microstructure has a rectangular cross section and a uniform saturation magnetization. Changes to either of these assumptions induce variation in the internal magnetic fields along the thickness direction that can lead to a linear mode spacing over several modes. This is consistent with what we know of the CVD growth of V[TCNE]$_x$. For instance, the growth of V[TCNE]$_x$ in bar-shaped microstructures as used here leads to tapering at the edges [4]. Additionally, the CVD growth environment of V[TCNE]$_x$ experiences changes as the growth proceeds, which may lead to variations in the saturation magnetization in the thickness direction. This latter effect is not well-characterized.

Theoretically, a surface inhomogeneity as small as $\lambda_{\text{ex}}/\sqrt{\pi} \approx 12$ nm (V[TCNE]$_x$ exchange length) may cause a decrease in the saturation magnetization at the top and bottom of the film, leading to the lower-order magnon modes to be pinned and linearly dispersed [15, 16]. In particular, in Ref. [16] the author shows that the linear dispersion is nearly independent of the exact distribution of M_s. We further complement those results with a micromagnetic simulation [17] that reveals interesting features when the saturation magnetization has a

Kittel mode

$g_0/2\pi$	$\kappa_{m0}/2\pi$	$\mu_0 M_{eff}$
80 MHz	30 MHz	53.6 mT

LC resonator

$\kappa_{ext}/2\pi$	$\kappa_r/2\pi$	$\omega_r/2\pi$	ϕ
0.2 MHz	0.902 MHz	3.58 GHz	-0.5 rad

Thickness quantized thickness magnon modes

	$g_n/2\pi$	$\omega_{mn}/2\pi$	$\kappa_{mn}/2\pi$
$n = 1$	30 MHz	$\omega_{m0}/2\pi + 26$ MHz	6 MHz
$n = 2$	15 MHz	$\omega_{m0}/2\pi + 46$ MHz	5 MHz
$n = 3$	8 MHz	$\omega_{m0}/2\pi + 64$ MHz	4 MHz
$n = 4$	3 MHz	$\omega_{m0}/2\pi + 79$ MHz	3 MHz
$n = 5$	1.5 MHz	$\omega_{m0}/2\pi + 98$ MHz	2 MHz
$n = 6$	1 MHz	$\omega_{m0}/2\pi + 113$ MHz	1 MHz

TABLE I. Parameters used to produce Fig. S4(b)

parabolic distribution that vanishes at the top and bottom. Along the thickness direction, the magnon excitations are dominated by the exchange interaction, therefore, for simplicity, we have turned off the demagnetization field from the simulation. In Fig. S5 we compare the case of uniform against non-uniform M_s. Fig. S5(a) shows the magnetic dispersion along the thickness direction for a cross section of the V[TCNE]$_x$ film, with dimensions 300 nm $\times$ 6 μm, where M_s is uniform. Fig. S5(d) shows an equivalent graph for a parabolic spatial distribution of M_s. First, the quantization is clear and, from Fig. S5(b), we checked a good agreement with the theory (Eq. (S2)) for the lower modes, with a small deviation at higher modes. Second, the non-uniform M_s generates more excitations than the perfect pinning boundary conditions. Third, and the most important conclusion from the simulation, a non-uniform M_s spreads the excitations over k space leading to $k = 0$ contributions for higher frequency modes, and that will enhance the coupling strength of higher modes with the resonator. We noticed that the non-uniform M_s led to different pinning conditions than the

uniform case (not shown), and we checked that simply enforcing the same pinning condition to the uniform case could not generate $k = 0$ contributions at higher frequencies, hence that is solely due to non-uniform M_s. Figs. S5(b) and S5(e) show the corresponding density of excitations (ρ) for each case.

In particular, Fig. S5(e) shows the appearance of new peaks as well as a shift from the result of Eq. (S2). This deviation, indicated by the difference between the peak locations and the dashed lines representing the solutions of Eq. (S2), seems to provide more spacing at lower frequencies and less at higher frequencies; this is consistent with approximately linearly spaced excitations. Another interesting feature is the broadening of the density near the Kittel mode because the modes are lying too close to each other. That could explain the broader signal for $\omega_{\pm}$ in the experiment, while the higher modes have a much narrower linewidth. Finally, Figs. S5(c) and S5(f) show the density at $k = 0$ (notice that $\rho_{k=0} \equiv m_y(k = 0)$). The spacing between these peaks appears even closer to linear than in Fig. S5(e). We expect those peaks to reflect the coupling strength to the resonator, thus the decay of the peaks intensities suggest a decreasing coupling to the resonator as identified by the fitting in Table I. We thus note that the very precise details of these minor modes in the spectrum are likely connected to complex features associated with the precise shape of the material and not central to the major report of strong coupling we provide in this work.

Another potential reason for deviation from the toy model is the presence of the superconducting inductor under the V[TCNE]$_x$ microstructure. The net precessing dipole moment of these magnon modes will produce dissipationless eddy currents in the superconducting wire that will also act back on V[TCNE]$_x$, causing an effective easy-plane anisotropy that will shift the magnon mode frequency. The higher the order of the $k \neq 0$ modes, the less eddy current induced because the smaller net precessing moment, and thus the smaller change in the frequency shift. Such effects are interesting but have not been studied extensively, and will be the topic for future work.

We now address the magnon modes that appear at frequencies less than the uncoupled uniform magnon mode frequency, which are generally attributed to backwards volume modes [18, 19]. Forward volume modes and surface spin waves have higher frequencies than the uniform magnon mode [20], so we do not consider them here as potential sources for these magnon modes below the uniform mode frequency. Our experimental geometry makes the excitation and detection of quantized backwards volume modes unlikely because the mag-

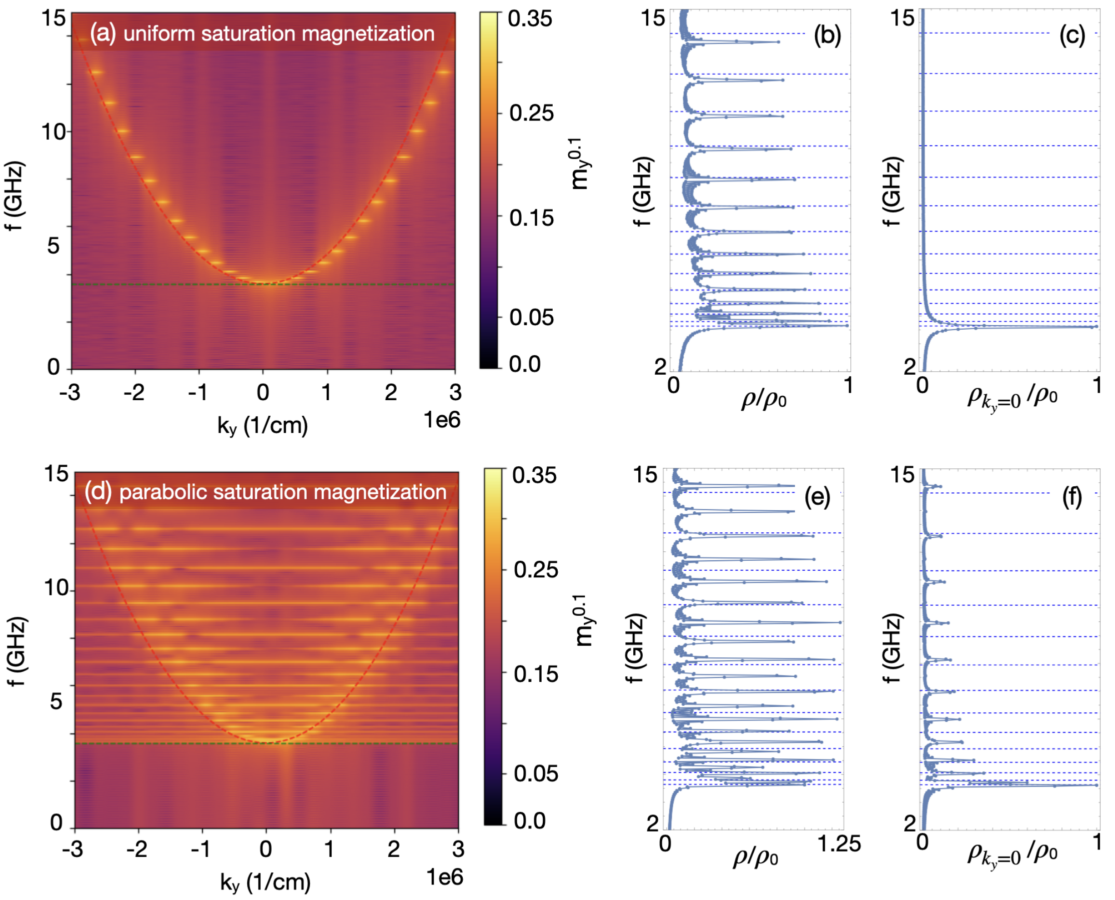

FIG. S5. 2D micromagnetic simulation for a cross section of the V[TCNE]$_x$ film, with dimensions 300 nm × 6 μm, and a uniform external magnetic field $B_0 = 0.128$ T applied along the long axis. Colors in (a) and (d) show the $m_y(k_y, f)$ component of the magnetization oscillating with frequency f and wavevector k_y along the thickness direction. The red-dashed curve follows Eq. (S2) for a continuous k_y and the green-dashed line marks the Kittel frequency $\gamma B_0/2\pi \approx 3.6$ GHz. (b) and (e) show the corresponding spinwave densities (ρ) to the left plots, normalized by the density at the Kittel frequency (ρ_0). (c) and (f) show the spinwave densities at $k_y = 0$, i.e., a cut on panels (a) and (d) at $k_y = 0$. The blue-dashed lines in (b), (c), (e), and (f) are the quantized frequencies ω_n from Eq. (S2) with $n = 0, 1, 2, \ldots$.

netic field is applied parallel to the long axis of the magnetic material, with an estimated misalignment of less than 3°. Because backwards volume modes require a wavevector parallel to the magnetization, this would require quantization along the (600 μm) long axis and cannot explain the magnon modes below the uniform mode frequency because this scenario

cannot impose visible quantization. We also note that the cross section of the sample is not rectangular at the edges due to the growth process [4], which in some situations could give rise to a nonuniform demagnetization field. However, this mechanism would also require a substantial magnetic field perpendicular to the long axis of the magnet, and is ruled out by our misalignment estimate above. Finally, we have also considered the possibility of a nonlinear process in which a resonator photon couples to the uniform mode magnon and induces a transition from a uniform mode magnon into a $k \neq 0$ magnon. This scenario is consistent with the appearance of modes at frequencies below the uncoupled uniform magnon mode, however, our estimate of the resonator photon and magnon populations suggests that such a nonlinear process would be orders of magnitude smaller than observed. Due to these inconsistencies with respect to typical explanations for magnon modes below the uniform magnon mode, we cannot make a definitive assignment of the lower frequency quantized modes.

VII. SECOND DEVICE RESULTS

To check the reproducibility of the magnon mode signals, we fabricate and measure a second V[TCNE]$_x$ resonator device. While the nominal aspects of the experiment are identical to the one discussed in the main text, a few details have changed. Specifically, the shape and pattern of the V[TCNE]$_x$ are the same as that in the main text, and the resonator pattern is also the same. However, this time we use reactive ion etching (RIE) for Nb etching and the V[TCNE]$_x$ was encapsulated with a different brand of epoxy. The nominal Nb thickness is 50 nm. The V[TCNE]$_x$ was again grown using CVD, however, it was grown in a new reactor. With the encapsulated V[TCNE]$_x$, the LC resonator's frequency $\omega_r/2\pi = 3.7264$ GHz and $Q_l = 7870$ at $B_0 = 0$.

Fig. S6(a) shows the microscope image of this device, and Fig. S6(b). Fig. S6(c) and (e) show S_{21} measured as a function of B_0 and ω at 0.44 K over narrowed ranges of magnetic field and frequency. We use -75 dBm of microwave power at the sample. In Fig. S6(c) we see three significant avoided crossings (at 0.105 T, 0.109 T and 0.113T) that correspond to at least three Kittel modes with different resonance frequencies that are each coupled to the LC resonator. This effect is likely caused by inhomogeneous anisotropy of the V[TCNE]$_x$ microstructure, where different spacial regions have a slightly different resonance field, likely due to an inhomogeneous strain induced in from differently thermal expansion

at cooldown. Ref. [5] describes that substrate-induced strain attributed to the mismatch of thermal expansion coefficients can introduce large shifts in the anisotropy. This could originate from either the substrate or the encapsulating epoxy.

In addition to the strong coupling of Kittel modes that result in three avoided crossings, we see thin diagonal stripes in $\Delta|S_{21}|$ that originate from higher-order magnon modes, as discussed above and in the main text. We again use the input-output theory to simulate Fig. S6(c). Because of this sample's homogeneous anisotropy, there are many more high-order magnon modes present in the spectrum than shown in the main text sample. These modes hybridize with the resonator modes and the Kittel modes, and as a result, it is not feasible to accurately index each magnon mode, fit the data, and extract the set of κ_{mn} and g_n accurately. However, it is clear that these high-order magnon modes have a much lower damping rate than the Kittel modes and that their coupling rate to the LC resonator is much less comparing to the Kittel modes, as before. Fig. S6(d) shows a simulation with qualitatively selected parameters. We use a 70 MHz linewidth for all three Kittel modes and a 1 MHz linewidth for all the high-order magnon modes. This model qualitatively reproduces the data and is consistent with the discussion in the main text showing that the high-order magnon modes have much smaller damping rates than the Kittel modes.

To examine the thickness quantized magnon mode linewidths more carefully, Fig. S6(f) shows a frequency linecut (red dashed line in (e)) of a high-order magnon mode with an associated fit to determine the linewidth. The fitted linewidth is 3.44(6) MHz. This linewidth is larger than the pure magnon mode linewidth because it is partially hybridized with the Kittel mode. The contribution of the LC resonator to this linewidth is negligible because this fitting shows $Q_c = 1.39 \times 10^6$, while the bare LC at $B_0 = 0$ has $Q_c = 1.06 \times 10^4$.

VIII. TEMPERATURE DEPENDENCE

Using the techniques discussed in the main text, we also investigate properties of our hybrid resonator-magnon (the sample discussed in the main text, see Fig 1(a)) at higher temperatures. We plot g and κ_m in Fig. S7(a) and S7(b) respectively. We see that g decreases and κ_m increases with increasing temperature.

IX. RESULTS FROM A HIGHER FREQUENCY LC RESONATOR

Other than the 3.6 GHz resonator as described in the main text, we also study another resonator-magnon device that is designed with a larger resonance frequency. It has the same inductor wire dimensions, but a smaller capacitance. The device resonance frequency is around 9.2 GHz, measured at zero field after V[TCNE]$_x$ deposition and encapsulation. A microscope image of the device after V[TCNE]$_x$ deposition is shown in Fig. S8(a). In this device there is incomplete lift-off. A large area of V[TCNE]$_x$ remains on the device, which is connected to the V[TCNE]$_x$ strip on the inductor wire. This may result in the V[TCNE]$_x$ strip coupled to a continuum of magnon modes.

Fig. S8(b) shows $\Delta|S_{21}|$ as a function of magnetic field and frequency acquired at 0.43 K and -65 dBm of microwave power. As with the device discussed in the main text, we verify that we operate the device in the linear response regime; here between -55 dBm to -65 dBm. We again see a strong avoided level crossing, indicating that this device operates in the strong coupling regime. Both κ_r and κ_m are larger as compared to the 3.6 GHz device, and the upper and lower branch resonance signals are weaker at $B_0 = B_{res}$. We fit the data using equation (2) of the main text to extract ω_+, κ_+ from 0.249 T to 0.291 T, and ω_-, κ_- from 0.299 T to 0.344 T. As in the main text, we then substitute $\omega_\pm$ into equation (3) of the main text and treat g, ω_{r0}, γ_r and M_{eff} as free parameters. We obtain $g/2\pi = 147.21(29)$ MHz and $\mu_0 M_{\text{eff}} = 72.40(11)$ mT, $\omega_{r0}/2\pi = 9.2529(15)$ GHz and $\gamma_r/2\pi = -71.7(5.1)$ MHz/T. Also, assuming linear dependence of κ_r on B_0, we evaluate $\kappa_+(B_0 = 0.249 \text{ T}) \approx \kappa_r(B_0 = 0.249 \text{ T})$ and evaluate $\kappa_-(B_0 = 0.344 \text{ T}) \approx \kappa_r(B_0 = 0.344 \text{ T})$. We find $\kappa_r(B_{res})/2\pi = 7.917(15)$ MHz.

To extract κ_m, we use [21]

$$\tilde{\omega}_\pm = \tilde{\omega}_r + \tilde{\Delta}/2 \pm \sqrt{\tilde{\Delta}^2 + 4g^2}/2, \tag{S3}$$

where $\tilde{\omega} = \omega - i\kappa/2$ and $\tilde{\Delta} = \tilde{\omega}_m(B_0) - \tilde{\omega}_r$. The real part of this equation gives equation (3) when $\omega_{r,m} \gg \kappa_{r,m}$, and the imaginary part gives

$$\kappa_\pm = [\kappa_r/2 + \kappa_m/2 \mp \text{Im}\sqrt{(-\omega_r + i\kappa_r/2 + \omega_m - i\kappa_m/2)^2 + 4g^2}]. \tag{S4}$$

Using this expression of $\kappa_\pm$, and treating κ_m, M_{eff} and g as free parameters, we obtain $\kappa_m/2\pi = 115$ MHz. The data and the fit are shown in Fig. S8(c). Therefore, this 9.2 GHz

resonator-magnon hybrid system also operates in the strong coupling regime with $\mathcal{C} = 93.0(4.2)$. For the same system at 3.0 K, we extract $\mu_0 M_{\text{eff}} = 72.94(10)$ mT, $\kappa_r(B_{res})/2\pi = 10.456(31)$ MHz, $\kappa_m/2\pi = 112.9(4.8)$ MHz, $g/2\pi = 146.20(26)$ MHz. Therefore the cooperativity is $\mathcal{C} = 72.4(3.2)$.

As in the main text, we measure $\Delta|S_{21}|$ with higher resolution around the avoided level crossing at 0.43 K [Fig. S8(d)]. We also see a faint $k \neq 0$ magnon mode within the avoided-crossing gap between $\omega_{\pm}$.

X. MAGNETIC FIELD ALIGNMENT

In our setup, the sample was placed such that the direction of the static field is parallel to the inductor wire of the LC resonator. However, there can be some misalignment caused by the variation of varnish thickness underneath the chip and the rotation of printed circuit board (PCB) on the cold finger. We estimated the upper limit of both in plane misalignment (causing static field in width direction of V[TCNE]$_x$) and out of plane misalignment (causing static field in thickness direction of V[TCNE]$_x$) to be 3 degree. Note that the out-of-plane component of static field will cause vortices in the inductor wire of the LC resonator.

XI. BACKGROUND TRANSMISSION

The background transmission $S_{21,0}(\omega)$ originates from voltage reflections in the transmission line that creates frequency dependent standing waves. Therefore, it depends on ω and has a very weak or no B_0 dependence. We measure it at values of B_0's and ω that are far from the upper or lower branch (see Fig. 2(a), 2(b)). In Fig. 2(a) for the 3.6 GHz device, $S_{21,0}(\omega)$ is measured from 3.48 GHz to 3.595 GHz at 0.1024 T, and from 3.595 GHz to 3.71 GHz at 0.1064 T. In Fig. 2(b), $S_{21,0}(\omega)$ is measured from 3.395 GHz to 3.495 GHz and from 3.595 GHz to 3.795 GHz at 0.1071 T, and 3.495 GHz to 3.595 GHz at 0.1017 T. Because $S_{21,0}(\omega)$ depends weakly on B_0, in Fig. 2(b) we added a DC offset of $|S_{21}|$ from 3.395 GHz to 3.495 GHz and from 3.595 GHz to 3.795 GHz such that $\Delta|S_{21}|$(3.495 GHz, 0.1017 T) = $\Delta|S_{21}|$(3.495 GHz, 0.1071 T) and $\Delta|S_{21}|$(3.595 GHz, 0.1017 T) = $\Delta|S_{21}|$(3.595 GHz, 0.1071 T) to stitch the 3 frequency ranges.

Similarly, in Fig. S8(b) for the 9.2 GHz device, $S_{21,0}(\omega)$ is measured from 8.9 GHz to

9.25 GHz at 0.2913 T, and from 9.25 GHz to 9.6 GHz at 0.3021 T. In Fig. S8(d), $S_{21,0}(\omega)$ is measured from 8.63 GHz to 9.23 GHz and from 9.47 GHz to 9.83 GHz at 0.2886 T, and 9.23 GHz to 9.47 GHz at 0.3021 T. Again, in Fig. S8(d), we added a DC offset of $|S_{21}|$ from 8.63 GHz to 9.23 GHz and from 9.47 GHz to 9.83 GHz such that $\Delta|S_{21}|$(8.63 GHz, 0.2886 T) = $\Delta|S_{21}|$(8.63 GHz, 0.3021 T) and $\Delta|S_{21}|$(9.23 GHz, 0.2886 T) = $\Delta|S_{21}|$(9.23 GHz, 0.3021 T) to stitch the 3 frequency ranges.

XII. KERR NON-LINEARITY

It is useful to assess the intrinsic Kerr nonlinearity in this cavity-magnonic system. Based on the resonator characterization discussed in Sec. IV, we note that the resonator nonlinearity is negligible. From equation (B8) in [22], the frequency of magnon is: $\omega_m^{'} = \omega_m + K * n$, where ω_m is the Kittel mode frequency in the limit of low power, n is the magnon number and K accounts for the Kerr non-linearity which is caused by the magnetic anisotropy. Fig. S9(a) is acquired using the same V[TCNE]$_x$ sample used in the main text. The microwave power is -85 dBm. From that we extracted the frequency scan near the lower branch resonance frequency ω_- at 0.10519 T (as marked by the red dashed line) and then extract ω_-, where

$$\omega_- = \frac{\omega_r}{2} + \frac{\omega_m^{'}}{2} - \frac{\sqrt{(\omega_m^{'} - \omega_r)^2 + 4g^2}}{2} = \frac{\omega_r}{2} + \frac{\omega_m + K * n}{2} - \frac{\sqrt{(\omega_m + K * n - \omega_r)^2 + 4g^2}}{2}$$

In this case, $\omega_m + K * n - \omega_r << 4g^2$, we have $\sqrt{((\omega_m + K * n - \omega_r)^2 + 4g^2)} \approx$ constant. So: $\omega_- = 2\pi C + Kn/2$, where C is a constant. Then we repeat the frequency sweep at higher powers, plotted ω_- vs calculated magnon number, and fit the result with $\omega_-/2\pi = C + Kn/4\pi$ (Fig. S9(b)). From this we extract $K/2\pi = -21.0(5)$ Hz. The error analysis does not include uncertainty in the absolute microwave power at the device.

XIII. RELATIONSHIP BETWEEN GILBERT DAMPING AND MAGNON LINEWIDTH

Understanding the relationship between homogeneous damping that is typically parameterized in terms of the phenomenological Gilbert damping coefficient, α and the magnon (ferromagnetic resonance, FMR) linewidth is important for designing future magnon-based

quantum systems. This is a topic that is not fully understood for very low temperature measurements, and while full elucidation is beyond the scope of the current work, here we discuss what is known and compare with broadband FMR measurements.

We describe the contributions to magnon linewidth in terms of magnetic field linewidth in accordance with the FMR literature. They can be related to frequency linewidth contributions $\Delta\kappa$ through the gyromagnetic ratio γ using $\Delta H = \Delta\kappa/\gamma$. The total linewidth is given as the sum of contributions,

$$\Delta H_{tot} = \frac{2\alpha}{\gamma}\omega + \Delta H_{TLS} + \Delta H_{TMS} + \Delta H_0, \tag{S5}$$

where the first term represents Gilbert damping with coefficient α. The second term is the damping caused by the coupling of the magnon mode to two-level system impurities and is discussed further below. The third term is the damping caused by magnon scattering, which for small amplitude excitations may include two-magnon scattering in the presence of spatial defects such as voids in the film. The last term represents inhomogeneous broadening caused by the spatial variation of the total anisotropy field.

Two-magnon scattering is allowed when the static magnetic field is applied in-plane[23] as in our experiment. In this process, uniform $k = 0$ mode magnons are scattered to $k \neq 0$ modes with degenerate energy. Momentum matching is provided by the spatial frequency introduced by spatial defects in the magnetic film. However, two-magnon scattering won't happen for out-of-plane magnetic field since there is no $k \neq 0$ magnon mode with energy degenerate with the uniform magnon mode.

XIV. MAGNON LINEWIDTH CONTRIBUTIONS DUE TO TWO-LEVEL SYSTEMS (TLS) COUPLING

Here we present an existing TLS model for V[TCNE]$_x$ from Yusuf et al. [5] and make a comparison with our measurements of $\kappa_m(T)$ presented in Fig. S7. Because we only measured at four temperatures, any fitting here is purely to show that the TLS model is consistent with the temperature dependence, not to assert uniqueness of the parameters or that it fully explains the data.

The TLS contribution to field linewidth can be written as [5]:

$$\Delta H_{TLS} = \Gamma_{TLS}(T) \tanh\left(\frac{1}{2}\frac{\hbar\omega}{k_B T}\right) \tag{S6}$$

Parameter	Ref. [5] Value	This work
S	1	1
τ	1	1
E_b	1 meV	0.257±0.056 meV
β	36.5 GHz	9.7 ±3.5 GHz

TABLE II. Parameters used in the TLS model, taken from Ref. [5].

where

$$\Gamma_{TLS}(T) = \frac{S}{\gamma}\left(\frac{N_{imp}}{N}\omega_{int}\right)\frac{\omega_{int}t_{\infty}e^{E_B/k_BT}}{1+(\omega-\omega_{eg})^2t_{\infty}^2e^{2E_b/k_BT}}. \tag{S7}$$

The variables and parameters are all discussed in Ref. [5]. $\hbar\omega_{int}$ is an impurity energy level and $\hbar\omega_{eg}$ refers to the energy separation between the majority and minority states. Ref. [5] takes $\omega_{eg} \sim \omega_{int}$. A useful parameterization is to let $\beta = \frac{N_{imp}}{N}\omega_{eg}$ and $\tau = \omega_{eg}t_{\infty}$. Also, in Ref. [5] it was suggested that $\hbar\omega_{eg} \sim 1.3$ meV, which means that $(\omega-\omega_{eg})^2 \sim \omega_{eg}^2$ for the frequency ranges of our experiment. So we can use

$$\Delta H_{TLS} = \frac{S}{\gamma}\frac{\beta\tau e^{E_b/k_BT}}{1+\tau^2e^{2E_b/k_BT}}\tanh\left(\frac{1}{2}\frac{\hbar\omega}{k_BT}\right) \tag{S8}$$

to plot and get a sense of the functional form. The parameters used in Ref. [5] are shown in Table II, and Eqn. S8 is plotted in Fig. S10. We vary model parameters to demonstrate how each one influences the temperature dependence of ΔH_{TLS}, shown in Fig. S11.

To see whether Eqn. S8 is consistent with the measured temperature dependence of κ_m, we fit to the data shown in Fig. S7(b). We allow E_b and β to vary as free parameters, and get the result shown in Fig. S12. The fitted parameters are shown in Table II. We stress that the purpose of this fit is merely to show that the temperature dependence is consistent with a TLS mechanism in V[TCNE]$_x$, and we do not regard the resulting parameters as unique. Understanding how the parameters depend on growth, environmental conditions, and frequency is an important topic that will be the subject of future work.

XV. FITTING FOR FIGURE 2(d)

According to input-output theory, the transmission spectrum of our coupled system composed of a microwave resonator and a uniform magnon mode can be modeled as [24]:

$$\Delta|S_{21}|(\omega) = \left|1 + \frac{(\kappa_{ext}/2)e^{-i\phi}}{i(\omega-\omega_{res}) - \kappa_r/2 + \frac{g^2}{i(\omega-\omega_{res})-\kappa_m/2}}\right| \tag{S9}$$

where ϕ is added to include the phase of the coupling Q [8]. Equation (S9) is a good model of the data only if the two dips at B_{res} have the same phase ϕ. However, in Fig. 2(d) the two dips have different phases: one dip is symmetric and the other is not. The plot of ΔS_{21} in the complex plane also shows the different phases of these two resonances. A circulator was not installed in the cryostat when we measured this sample, so the different phases are caused by frequency-dependent standing waves in the transmission line that is coupled to the LC resonator. As a result, it is not appropriate to fit Eqn. S9 to the data shown in Fig. 2(d). Instead, we fit the two dips separately using main text equation (2). This approach is appropriate in the limit $\kappa_\pm \ll 2g \ll \omega_\pm$, as is the case for our experiment where $2\pi \times 16$ MHz $\ll 2\pi \times 180$ MHz $\ll 2\pi \times 3.5$ GHz.

[1] J. T. Hou and L. Liu, Strong coupling between microwave photons and nanomagnet magnons, Physical Review Letters **123**, 107702 (2019).

[2] C. Eichler, A. Sigillito, S. A. Lyon, and J. R. Petta, Electron spin resonance at the level of 10000 spins using low impedance superconducting resonators, Physical Review Letters **118**, 037701 (2017).

[3] A. H. Trout, S. W. Kurfman, Y. Shi, M. Chilcote, M. E. Flatté, E. Johnston-Halperin, and D. W. McComb, Probing the structure of vanadium tetracyanoethylene using electron energy-loss spectroscopy, APL Materials **10**, 081102 (2022).

[4] A. Franson, N. Zhu, S. Kurfman, M. Chilcote, D. R. Candido, K. S. Buchanan, M. E. Flatté, H. X. Tang, and E. Johnston-Halperin, Low-damping ferromagnetic resonance in electron-beam patterned, high-Q vanadium tetracyanoethylene magnon cavities, APL Materials **7**, 121113 (2019).

[5] H. Yusuf, M. Chilcote, D. R. Candido, S. Kurfman, D. S. Cormode, Y. Lu, M. E. Flatté, and E. Johnston-Halperin, Exploring a quantum-information-relevant magnonic material: Ultralow damping at low temperature in the organic ferrimagnet V[TCNE]$_x$, AVS Quantum Science **3**, 026801 (2021).

[6] D. F. Santavicca, J. K. Adams, L. E. Grant, A. N. McCaughan, and K. K. Berggren, Microwave dynamics of high aspect ratio superconducting nanowires studied using self-resonance, Journal of Applied Physics **119**, 234302 (2016).

[7] M. R. Vissers, J. Hubmayr, M. Sandberg, S. Chaudhuri, C. Bockstiegel, and J. Gao, Frequency-tunable superconducting resonators via nonlinear kinetic inductance, Applied Physics Letters **107**, 062601 (2015).

[8] S. Probst, F. Song, P. A. Bushev, A. V. Ustinov, and M. Weides, Efficient and robust analysis of complex scattering data under noise in microwave resonators, Review of Scientific Instruments **86**, 024706 (2015).

[9] A. Schneider, *Quantum Sensing Experiments with Superconducting Qubits* (KIT Scientific Publishing, 2021).

[10] A. G. Gurevich and G. A. Melkov, *Magnetization Oscillations and Waves*, 1st ed. (CRC Press, 1996).

[11] B. A. Kalinikos and A. N. Slavin, Theory of dipole-exchange spin wave spectrum for ferromagnetic films with mixed exchange boundary conditions, Journal of Physics C: Solid State Physics **19**, 7013 (1986).

[12] H. Puszkarski, P. Tomczak, and H. T. Diep, Surface anisotropy energy in terms of magnetocrystalline anisotropy fields in ferromagnetic semiconductor (Ga,Mn)As thin films, Physical Review B **94**, 195303 (2016).

[13] Q. Wang, B. Heinz, R. Verba, M. Kewenig, P. Pirro, M. Schneider, T. Meyer, B. Lägel, C. Dubs, T. Brächer, and A. V. Chumak, Spin pinning and spin-wave dispersion in nanoscopic ferromagnetic waveguides, Physical Review Letters **122**, 247202 (2019).

[14] Y. Tabuchi, S. Ishino, A. Noguchi, T. Ishikawa, R. Yamazaki, K. Usami, and Y. Nakamura, Coherent coupling between a ferromagnetic magnon and a superconducting qubit, Science **349**, 405 (2015).

[15] A. M. Portis, LOW-LYING SPIN WAVE MODES IN FERROMAGNETIC FILMS, Applied Physics Letters **2**, 69 (2004).

[16] M. Sparks, Ferromagnetic resonance in thin films. iii. theory of mode intensities, Phys. Rev. B **1**, 3869 (1970).

[17] A. Vansteenkiste, J. Leliaert, M. Dvornik, M. Helsen, F. Garcia-Sanchez, and B. Van Waeyenberge, The design and verification of Mumax3, AIP Advances **4**, 107133 (2014).

[18] G. B. G. Stenning, G. J. Bowden, L. C. Maple, S. A. Gregory, A. Sposito, R. W. Eason, N. I. Zheludev, and P. A. J. de Groot, Magnetic control of a meta-molecule, Optics Express **21**, 1456 (2013).

[19] B. Bhoi, T. Cliff, I. S. Maksymov, M. Kostylev, R. Aiyar, N. Venkataramani, S. Prasad, and R. L. Stamps, Study of photon–magnon coupling in a YIG-film split-ring resonant system, Journal of Applied Physics **116**, 243906 (2014).

[20] A. Serga, A. Chumak, and B. Hillebrands, YIG magnonics, Journal of Physics D: Applied Physics **43**, 264002 (2010).

[21] M. Harder, B. Yao, Y. Gui, and C.-M. Hu, Coherent and dissipative cavity magnonics, Journal of Applied Physics **129**, 201101 (2021).

[22] Y. P. Wang, G. Q. Zhang, D. K. Zhang, X. Q. Luo, W. Xiong, S. P. Wang, T. F. Li, C. M. Hu, and J. Q. You, Magnon kerr effect in a strongly coupled cavity-magnon system, Physical Review B **94**, ARTN 224410 10.1103/PhysRevB.94.224410 (2016).

[23] R. D. McMichael and P. Krivosik, Classical model of extrinsic ferromagnetic resonance linewidth in ultrathin films, IEEE Transactions on Magnetics **40**, 2 (2004).

[24] P. G. Baity, D. A. Bozhko, R. Macêdo, W. Smith, R. C. Holland, S. Danilin, V. Seferai, J. Barbosa, R. R. Peroor, S. Goldman, U. Nasti, J. Paul, R. H. Hadfield, S. McVitie, and M. Weides, Strong magnon–photon coupling with chip-integrated YIG in the zero-temperature limit, Applied Physics Letters **119**, 033502 (2021).

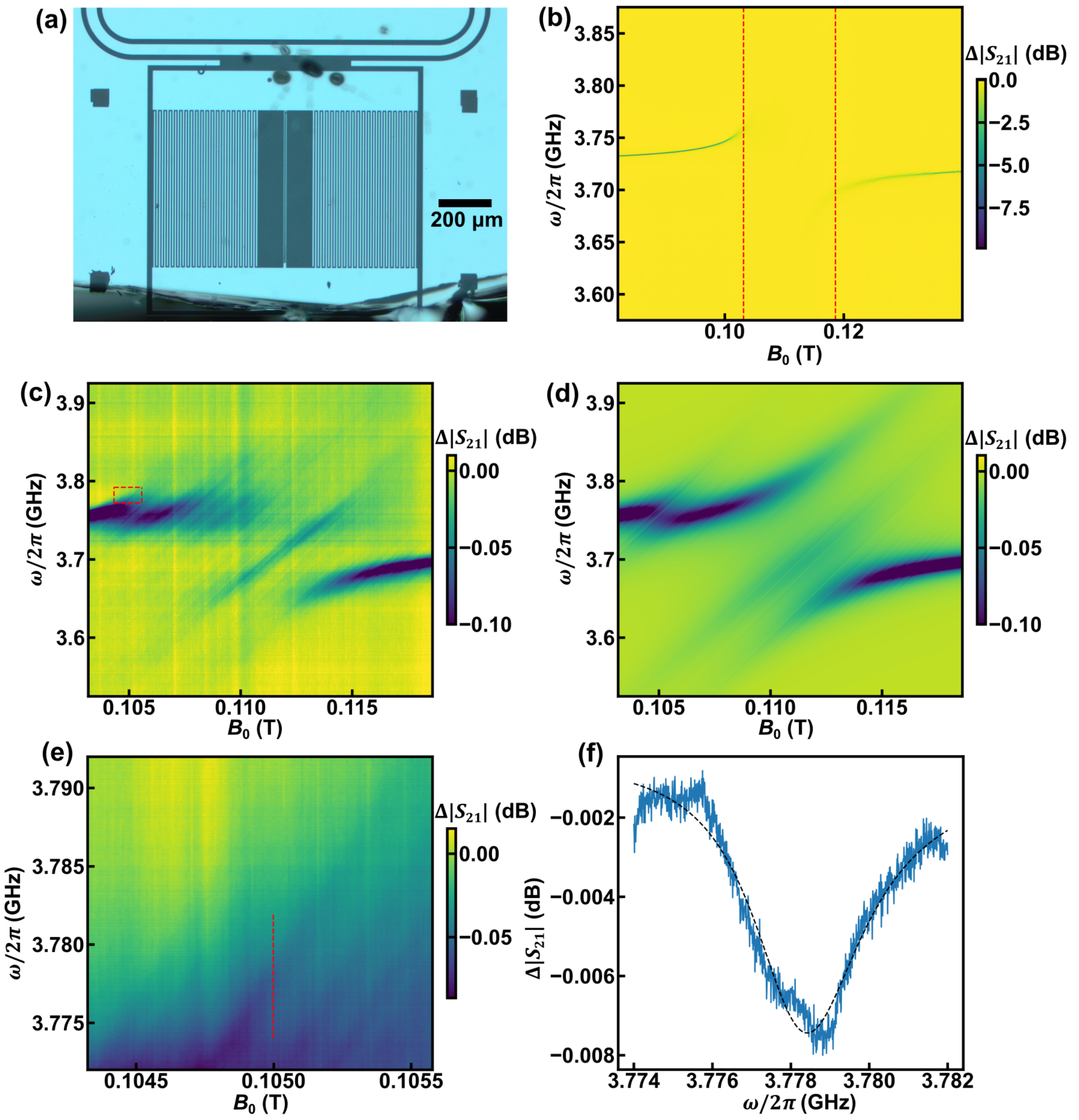

FIG. S6. Result of another sample of V[TCNE]$_x$ on a LC resonator. (a) Microscope image of the coupled system. (b) S_{21} measured as a function of B_0 and ω. (c) S_{21} measurements with finer steps near resonance field (between the red dashed lines in (b)). (d) Simulated transmission spectrum to qualitatively reproduce (c). In this simulation, all the three Kittel modes have linewidth of 70 MHz and all the higher order magnon modes have linewidth of 1 MHz. (e) S_{21} measurements with further finer steps to capture one of the magnon modes signal (inside the red dashed rectangle in (c)). (f) Frequency sweep linecut for measuring the linewidth of the magnon mode signal (red dashed line in (e)). The fitted linewidth is 3.44(6) MHz. This linewidth is bigger than this pure higher order magnon mode's linewidth because there is some Kittel mode contribution.

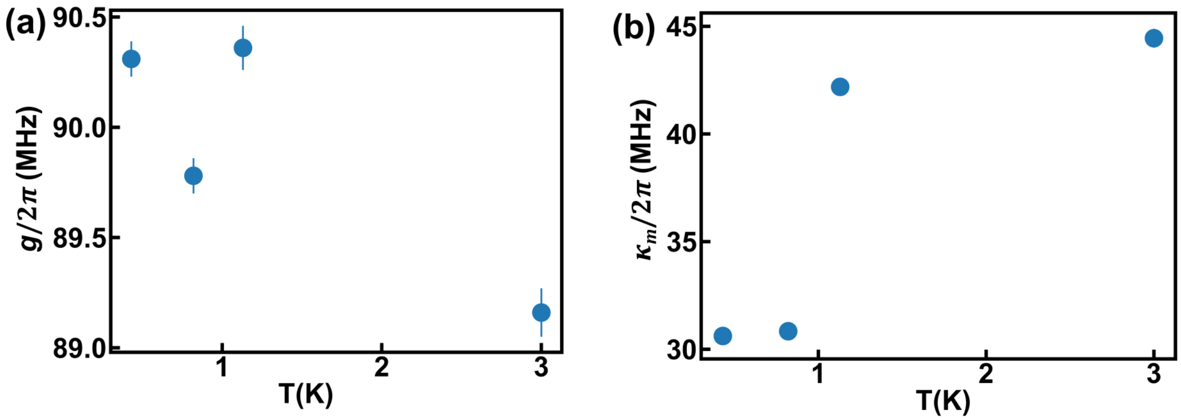

FIG. S7. MW measurement of V[TCNE]$_x$ coupled to the 3.6 GHz resonator at different temperatures. (a) Temperature dependence of g extracted from the S_{21} measurements at B_{res}, (b) Temperature dependence of κ_m extracted from S_{21} linewidth measurements at B_{res}. The error bars for κ_m are smaller than the circular point markers.

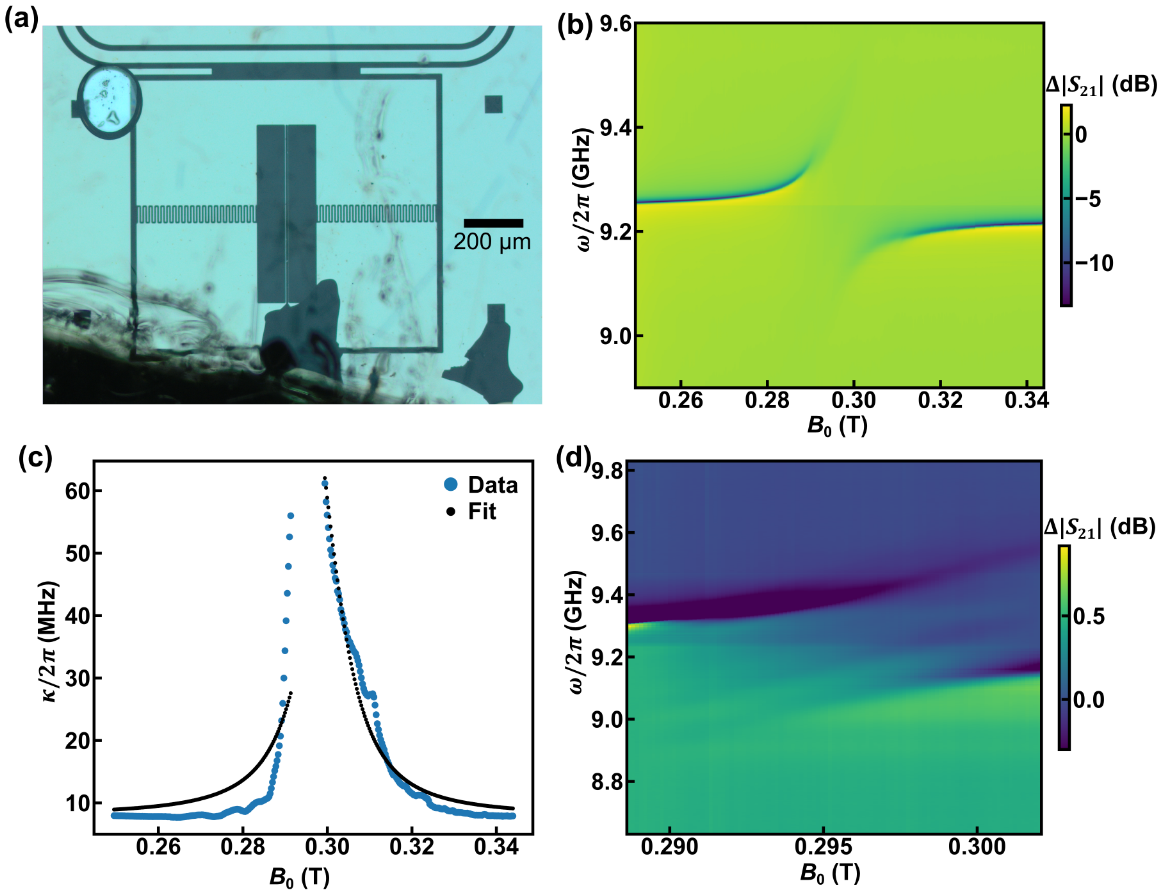

FIG. S8. MW measurement of V[TCNE]$_x$ coupled to the 9.2 GHz resonator at 0.43 K. (a) Microscope image of V[TCNE]$_x$ on the 9.2 GHz resonator. (b) S_{21} measured as a function of B_0 and ω. (c) Damping rate of the upper branch vs B_0 from 0.249 T to 0.291 T, and that of the lower branch from 0.299 T to 0.344 T. The error bars are smaller than the circular point markers. The fitted magnon damping rate is $\kappa_m = 117.7(5.3)$ MHz. (d) S_{21} measurements with finer steps in B_0 showing a $k \neq 0$ magnon mode around 150 MHz higher than the lower branch frequency.

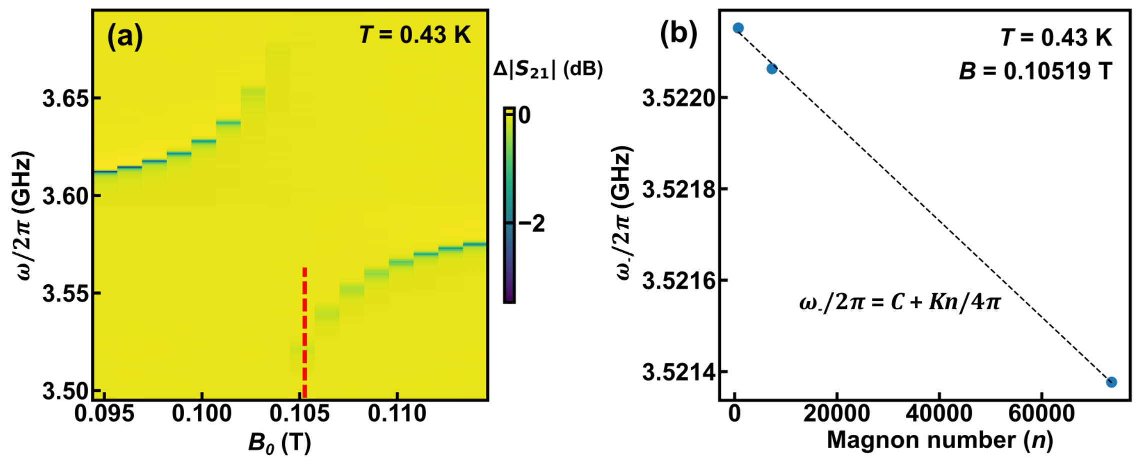

FIG. S9. (a) S_{21} measured as a function of B_0 and ω when the power at the sample is -85 dBm. (b) Lower branch resonance frequency vs magnon number (blue dots) and the fitting (black dashed line) to extract Kerr non-linearity K. The error bar of the data is too small to be seen on this scale.

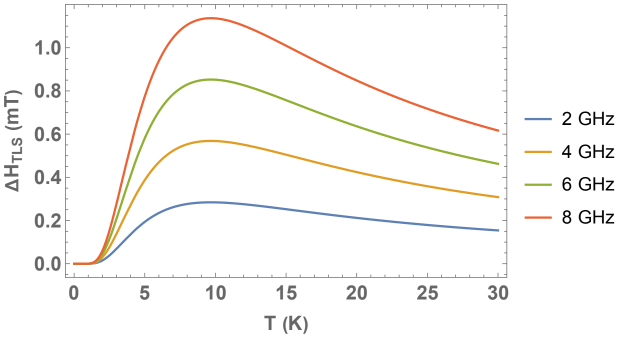

FIG. S10. Plot of ΔH_{TLS} using parameters in Table II for Ref. [5].

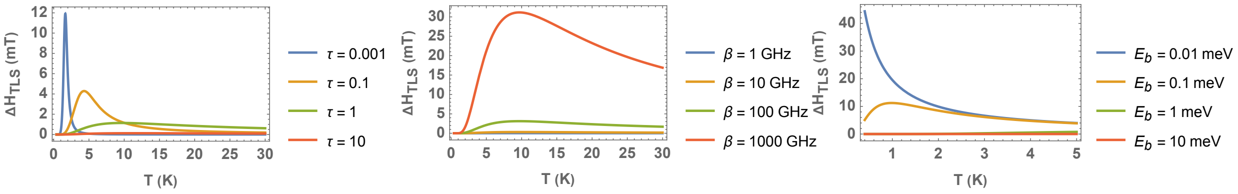

FIG. S11. Plot of ΔH_{TLS} by varying τ, β and E_b from Table II

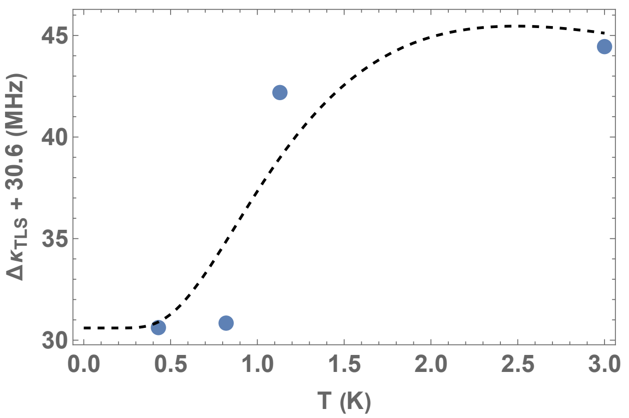

FIG. S12. A fit of $\kappa_m(T)$ data (blue points) to Eqn. S8 allowing E_b and β to vary as free parameters. A saturated linewidth of 30.6 MHz is also include so that only temperature variations are captured by the fit.

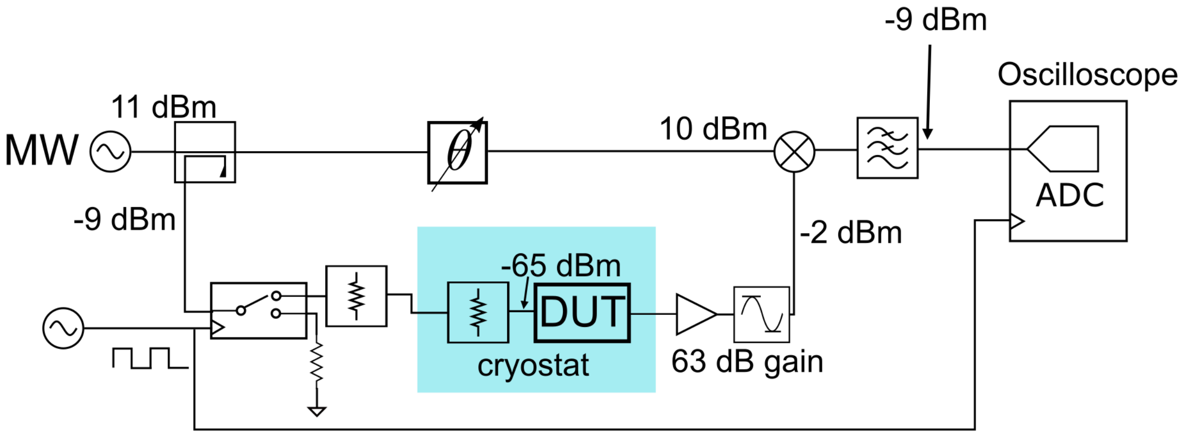

FIG. S13. Circuit diagram for the ring-down experiment.

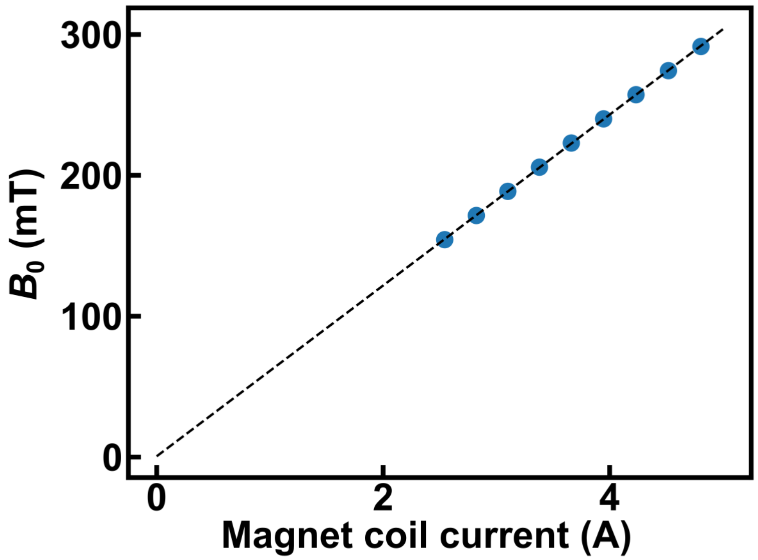

FIG. S14. Experimental data for the static field at the sample B_0 vs the magnet coil current (blue dots, with error bars are smaller than the circular dots) and the linear fit (black dashed line) to extract the calibration factor of 60.64(10) mT/A. The black dashed line extrapolates to the origin as expected.
